# Inflammation and Oxidative Stress in the Context of Extracorporeal Cardiac and Pulmonary Support

**DOI:** 10.3389/fimmu.2022.831930

**Published:** 2022-03-04

**Authors:** Sanaz Hatami, Joshua Hefler, Darren H. Freed

**Affiliations:** ^1^ Department of Surgery, University of Alberta, Edmonton, AB, Canada; ^2^ Canadian National Transplant Research Program, Edmonton, AB, Canada; ^3^ Department of Biomedical Engineering, University of Alberta, Edmonton, AB, Canada; ^4^ Alberta Transplant Institute, Edmonton, AB, Canada; ^5^ Department of Physiology, University of Alberta, Edmonton, AB, Canada

**Keywords:** inflammation, oxidative stress, extracorporeal life support, *ex-situ* organ perfusion, cardiac and pulmonary function

## Abstract

Extracorporeal circulation (ECC) systems, including cardiopulmonary bypass, and extracorporeal membrane oxygenation have been an irreplaceable part of the cardiothoracic surgeries, and treatment of critically ill patients with respiratory and/or cardiac failure for more than half a century. During the recent decades, the concept of extracorporeal circulation has been extended to isolated machine perfusion of the donor organ including thoracic organs (*ex-situ* organ perfusion, ESOP) as a method for dynamic, semi-physiologic preservation, and potential improvement of the donor organs. The extracorporeal life support systems (ECLS) have been lifesaving and facilitating complex cardiothoracic surgeries, and the ESOP technology has the potential to increase the number of the transplantable donor organs, and to improve the outcomes of transplantation. However, these artificial circulation systems in general have been associated with activation of the inflammatory and oxidative stress responses in patients and/or in the exposed tissues and organs. The activation of these responses can negatively affect patient outcomes in ECLS, and may as well jeopardize the reliability of the organ viability assessment, and the outcomes of thoracic organ preservation and transplantation in ESOP. Both ECLS and ESOP consist of artificial circuit materials and components, which play a key role in the induction of these responses. However, while ECLS can lead to systemic inflammatory and oxidative stress responses negatively affecting various organs/systems of the body, in ESOP, the absence of the organs that play an important role in oxidant scavenging/antioxidative replenishment of the body, such as liver, may make the perfused organ more susceptible to inflammation and oxidative stress during extracorporeal circulation. In the present manuscript, we will review the activation of the inflammatory and oxidative stress responses during ECLP and ESOP, mechanisms involved, clinical implications, and the interventions for attenuating these responses in ECC.

## Introduction

Extracorporeal circulation systems (ECC), have been an essential part of life-support in critically ill patients and cardiothoracic surgeries (1). During the recent couple of decades, this technology has been extended to *ex-situ* organ perfusion (ESOP) to improve the protection, availability, and assessment of the donor hearts and lungs. These technologies have, without a doubt, been revolutionary, leading to significant improvements in surgical treatments, patient management, and donated thoracic organ utilization (2, 3). However, its application has met some limitations as well, with systemic inflammation and oxidative stress being among the most important challenges. These reactions negatively affect patient outcomes in extracorporeal life support (ECLS) (4–6), and may negatively affect organ preservation in ESOP (7, 8). In this review, we discuss the development of inflammatory and oxidative stress responses in ECLS and ESOP, involved mechanisms, and interventions to attenuate these responses.

## History of Thoracic Organ-Oriented Extracorporeal Circulation

### Extracorporeal Life Support

The first experiments that would lead to the development of ECLS began with César Julien Jean Le Gallois in the early 19^th^ century, who showed a decapitated rabbit could be kept alive through pulmonary inflation using a syringe. Later on, Eduard Brown-Séquard successfully stimulated isolated extremities by syringe reperfusion with blood, oxygenated through agitation while in contact with air. These experiments, alongside the advances in technology, including a device capable of infusing blood under pressure (by Ludwig and Schmidt in 1868) and development of film and bubble oxygenators (by von Schroder in 1882, and Frey and Gruber in 1885), facilitated the development of extracorporeal perfusion (9–11). These advances not only improved organ perfusion devices as “tools for studying the organs”, but also helped in subsequent development of the heart lung machine (cardiopulmonary bypass, CPB) by Gibbon in 1953, which allowed open-heart surgeries that had not been feasible before (12). With the subsequent success of CPB in in 1950s and 60s, and further advances in bioengineering of oxygenators, such as introduction of silicone rubber membrane oxygenators that could support oxygenation for days rather than hours, the ECLS was also extended to extracorporeal membrane oxygenation (ECMO), designed to provide longer periods of support. It provides augmented oxygenation (venovenous, VV-ECMO) and/or cardiac output (venoarterial, VA-ECMO) in patients of all ages who suffer the conditions associated with cardiopulmonary failure. The application of ECMO has also expanded to extracorporeal cardiopulmonary resuscitation, and bridging to lung transplantation, although it has been most successful in treatment of newborns with severe respiratory failure (5, 13, 14).

### *Ex-Situ* Thoracic Organ Perfusion

After the pioneering work of isolated (*ex-situ*)-perfused heart method by Cyon in 1866, the method was further adopted for perfusion of mammal heart by Langendorff in 1895 (3). With the introduction of isolated working heart preparation less than a century later in 1967 by Neely and Morgan, *ex-situ* heart perfusion (ESHP) has been since widely utilized for studying the heart (15).

During the recent few decades, ESOP has emerged again for its potential to preserve donated hearts and lungs in a more physiologic setting, and offering a venue for assessment and potentially improvement of donor organs. This technology has enhanced organ preservation and viability assessment, and has facilitated transplantation of sub-optimal and extended-criteria hearts and lungs (2, 16). Still, the optimal *ex-situ* preservation of thoracic organs function and viability has been a matter of active research. Formation of edema and tissue injury, as well as diminished functional status of the organ during extended *ex-situ* perfusion periods, limit the optimal *ex-situ* preservation time, and create an obstacle for potential advantages of this preservation/evaluation method. Meanwhile, the implications of the artificial materials/surfaces used in *ex-situ* organ perfusion in development of cellular injury and stress has been mostly neglected (3, 17, 18).

## Similarities and Differences Between ECLS and ESOP Settings and Apparatuses

The apparatuses/settings used for ESOP share many aspects with ECLS, particularly with CPB system. All these systems rely on artificial, synthetic materials/components including pumps and oxygenators for supporting circulation and oxygenation. However, there are obvious differences between those, including exclusive, isolated perfusion of the procured organ in ESOP as opposed to systemic perfusion in ECLS, or lesser blood-air interface in ECMO (closed circuit) compared to CPB. The similarities and differences of ESHP with ECLS systems are summarized in **Table 1** (4, 5, 19, 20).

**Table 1:** Differences between extracorporeal life support techniques and *ex-situ* thoracic organ perfusion.

	ECMO	CPB	ESOP
Application of artificial materials/components	Yes	Yes	Yes
Connection to body	Connected	Connected	Non-connected
Duration	Days to weeks	Minutes to hours	Minutes to hours
Hemodilution	No	Yes	Yes
Anticoagulation	Low-dose heparin	High-dose Heparin	High-dose Heparin
Reversal of anticoagulation	No	Yes (protamine)	No
Hypothermia	No	Yes	Variable
Air-blood interface	No (closed-circuit)	Yes (there are some closed-circuit variants)	Yes
Pulsatility	Variable with mode	No	Variable (dependent on device)

ECMO, extracorporeal membrane oxygenation; CPB, cardiopulmonary bypass; ESOP, ex-situ thoracic organ perfusion.

## Inflammation and Oxidative Stress in ECLS

It is well-established that ECLS leads to systemic inflammation and oxidative stress yet, the extent of these phenomena, clinical significance, and successful therapeutic intervention is still a matter of debate, mostly due to the significant heterogeneity in study design (e.g., patient populations), and the conflicting results observed (5, 21). The highlighted studies on induction of inflammatory and oxidative stress responses related to ECLS are summarized in **Supplementary Table 1** (22–50). A strong body of evidence suggests that inflammation and oxidative stress occur early after initiation of ECLS and progress over time. However, there are studies showing minimal or no change in the markers of immune system activation during application of ECLS or a delayed response detectible either later during ECC, or after its termination. On the other hand, among several different markers (e.g. cytokines, markers of oxidative stress) that may be induced in ECLS, only a few of them such as tumor necrosis factor alpha (TNF-α), interleukin (IL)-8, and IL-6 have been shown to have a correlation with organ function and/or patient outcomes (51–54). Regardless, clinical and experimental studies mostly suggest that the systemic responses precede the tissue-specific induction of inflammation or oxidative stress at the institution of ECLS, and pre-existing comorbidities play a key role in exacerbation of these responses.

## Inflammation and Oxidative Stress in *Ex-Situ* Thoracic Organ Perfusion

Despite the considerable paucity of data, the few existing studies by our team and others **Supplementary Table 1** suggest that similar to ECLS, ESOP is also associated with inflammation and oxidative stress (19, 55, 56). The extent and implications of these reactions on donor organ viability in ESOP may be critical. Donor organs routinely endure various ischemic times, promoting inflammation and oxidative stress, which may be even more severe in extended criteria donations, such as donation after circulatory death (DCD). Moreover, the absence of *in vivo* mechanisms to replenish and refine blood components in the setting ESOP may make perfused organs more vulnerable to oxidative stress. The importance of this has been strongly demonstrated by studies reporting successful, extended 24-hour and 72-hour ESHP in animal models involving cross-circulation with a live animal (57, 58), and the recent report on improved pulmonary function of *ex-situ*-perfused human lungs with xenogeneic (porcine) cross-circulation (59).

The limited available studies of ESOP have mainly focused on mitigation of the inflammatory responses in DCD or static cold storage (SCS) hearts and lungs that have been subjected to significant warm or cold ischemic times respectively (60–62). Thus, they may already be facing severe degrees of ischemia/reperfusion injury (IRI) during subsequent *ex-situ* perfusion, masking the inflammation and oxidative stress related to ECC itself. In the recent experimental studies of ESHP and ESLP by our team in a porcine model, we observed that perfusion of healthy hearts and lungs, which had not experienced the insults related to brain death or circulatory death was also accompanied by significant induction of various inflammatory mediators such as various interleukins (ILs) including IL-1β, IL-6, IL-8, IL-18, and TNF-α and/or markers of oxidative stress such as oxidized low density lipoprotein (ox-LDL), and malondialdehyde (MDA) (19, 56, 63). Clarifying the effects of activation of these responses on the quality of donor organs and eventually the outcomes of transplantation, warrants more experimental and clinical studies.

## The Pathophysiology of Inflammatory and Oxidative Stress Responses During Extracorporeal Circulation

**Figure 1:**
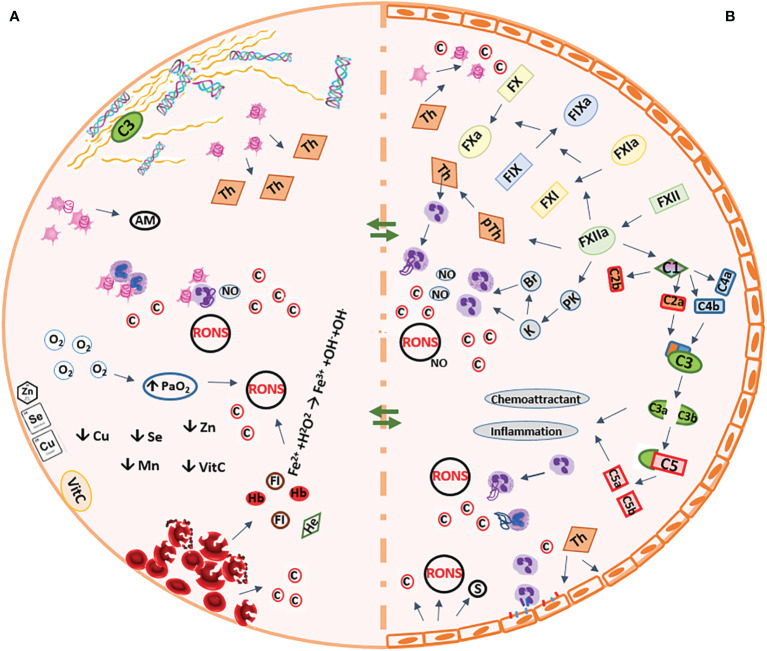
Summary of the pathophysiologic conditions occurring during extracorporeal circulation. **(A)** Artificial biomaterial of the circuit **(B)** Vascular system of the organs AM, adhesion molecules; Br, bradykinin; C, complement compartment, c, cytokines; Cu, copper; Fe^2+^, ferrous ion; Fe^3+^, ferric ion, FI, free iron; H_2_O_2_, hydrogen peroxide; He, heme; FIX, factor-9; FX, factor-10; FXI, factor-11; FXII, factor-12; K, kallikrein; Mn, manganese; NO, nitric oxide; O_2_, oxygen; OH**^-^**, hydroxyl; OH., hydroxyl radicalPaO_2_, partial pressure of arterial oxygen; PK, pre-kallikrein; pTh, prothrombin; RONS, reactive oxygen and nitrogen species; s, selectins; Se, selenium; Th, thrombin; VitC, vitamin C; Zn, zinc; 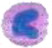, monocyte; 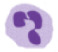, neutrophil; 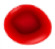, red blood cell.

The ECLS and ESOP systems, though different in many ways, all expose the body/organ to various types of non-physiological conditions, which may affect organ function and viability and patient outcomes (5, 21, 64). Various alterations are induced to elements of the blood/perfusate, as well as the exposed tissues/organs during ECC, which are discussed in the following sections and are also briefly reflected in **Figure 1**.

## Complement & Contact Activation System

According to the Vroman effect, within minutes after the contact between the blood and the artificial material (biomaterial) of the circuit, the sequential absorption of plasma proteins starts, with fibrinogen forming a surface for other plasma proteins to bind to the biomaterial. These proteins include but are not limited to the contact activation pathway molecules, albumin, and complement component protein 3 (C3). Formation of the protein layer on the biomaterial surface and activation of the complement system, boosts the interaction between the platelets and polymorphonuclear leukocytes (PMN) leading to release of different pro-inflammatory cytokines (65). Activation of the contact system leads to sequential cleavage of intrinsic coagulation pathway elements and activation of factors XII-X, leading to activation of intrinsic coagulation pathway and production of kallikrein and bradykinin (**Figure 1**). Increased concentrations of kallikrein and bradykinin can stimulate the release of cytokines and nitric oxide (NO) from the neutrophils and promote inflammation (5, 66). The activated factor X (Xa, common factor between intrinsic and extrinsic coagulation pathways) converts prothrombin to thrombin. Despite administration of heparin, thrombin is formed during extracorporeal circulation, and links coagulation with inflammation as it activates endothelial cells and induces production of ROS, selectins (on platelets and endothelial cells) and platelet activating factor, activates neutrophils and platelets and induces production and release of pro-inflammatory cytokines including IL-1α, IL-6, and IL-8 (5, 67, 68).

During ECC, activation of the complement system triggers inflammatory responses and increases capillary permeability and changes in vascular tone. The circulating products of complement system activation and cleavage, including cleaved compartments 3, 4, and 5 (C3a, C4a, and C5a, anaphylatoxins), can activate platelets and endothelial cells. Of the three initiating pathways of complement activation, the classical pathway (CP) and lectin pathway (LP) are induced by antigen-antibody complex formation, while the alternative pathway (AP) can be directly induced by the contact with the biomaterial of the circuit. The AP seems to be the main pathway activated during CPB, as a result of the contact of the blood with biomaterial of the circuit and cleavage of C3 (activation of the common complement pathway). However, the CP can also be activated during ECC due to the activation of the complement protein C1 by factor XIIa of the activated contact system, protamine-heparin complex formation (after administration of protamine at the end of CPB to prevent bleeding), and endotoxin released from the intestine during ischemia and reperfusion (6, 65).

Compared to CPB, there is a significant gap in the literature about the complement system activity during ECMO and its effects on the outcomes of the patients, despite the ECC times being usually longer in ECMO. Moreover, most of the existing literature are from the 1990s with much older technology (older versions of pumps and oxygenators). In the few existing, more recent studies on ECMO, a fairly rapid elevation in the concentrations of the complement system components has been reported (69, 70).

There is a distinct paucity of literature on the activation of the complement system related to *ex-situ* perfusion, despite these platforms being used to study the contribution of complement activation and inhibition in the pathophysiology and outcomes of IRI.

## Blood Cells and Endothelium

### Platelets

In 1995, Gemmell et al. showed that the contact of the blood with biosynthetic surfaces massively activated platelets, even in the presence of an anticoagulant (71). Platelet activation, triggered by tissue factor-induced thrombin, and carried on through the protease-activated receptors (PAR) on the platelet surface, leads to massive production of thrombin and thus further exacerbates the inflammatory response (65). In addition to thrombin as the main platelet activating factor, complement activation (through CP), and physical characteristics of the circuit also contribute to activation of platelets (5, 72).

Platelets, neutrophils, and endothelial cells interact mainly through CD40 on endothelial cell and CD40 ligand (CD40L) expressed on activated platelets. These interactions facilitate tissue migration of neutrophils and induce production, and release of chemokines and adhesion molecules in endothelial cells. Also, during ECC, the number of circulating, activated platelets bound to PMNs and monocytes increase, possibly triggering the pro-inflammatory effect of these immune cells (73, 74). Additionally, the activation of platelets may cause shape alterations leading platelets to release the contents of their granules into the circulation, including various chemokines such as chemokine (C-C motif) ligand 3, 5, 7, 17 and chemokine (C-X-C motif) ligand 4, 5, 7, and 8), pro-inflammatory cytokines such as IL-1β and CD-40 ligand, and adhesion molecules such as von Willebrand factor, and P-selectin, which can further promote the inflammatory response to ECC by other platelets, leukocytes and endothelial cells (5, 75).

### Red Blood Cells and Hemolysis

Particularly in smaller patients, ECLS involves transfusion of significant amounts of packed red blood cells (pRBCs) that may play a key role in induction of oxidative stress, particularly with aged pRBC, which typically contain lower levels of elements supporting the antioxidative defence of the cell, such as selenium (Se) (67). Hemolysis occurs inevitably, in a time-related fashion during ECC due to the blood passing through different artificial compartments of the circuit, including the oxygenator, pump, and reservoir, at varying speeds (76, 77).

Lysed RBCs release cell-free hemoglobin, haem and iron. Being potent damage association molecular patters (DAMPs), cell-free haemoglobin and haem will induce or exacerbate inflammatory responses and oxidative stress through triggering innate immunity, complement system, and endothelial cells contributing to end organ damage. Additionally, the Fenton reaction, converting haem-iron from a ferrous to ferric state, generates highly active hydroxyl radicals that further promote oxidative stress and related modifications to lipids and proteins, altering the cellular membrane polarity and permeability, making the cells more susceptible to lysis (67, 78, 79). Furthermore, RBCs release various pro-inflammatory cytokines and chemokines to the circulation as a result of hemolysis, or stress responses in intact cells. These inflammatory molecules include but are not limited to IL-1 family, TNF family, IL-6, interferon (INF)-α2 and INF-γ, and C-C motif chemokines (such as monocyte chemoattractant protein-1 (MCP-1), and MCP-3) and C-X-C motif chemokines (such as IL-8) (80).

Scavenging systems, including organs such as the liver and spleen that uptake and metabolise the haemopexin complex, monocytes and tissue macrophages that uptake haemoglobin-haptoglobin complex, and various antioxidative enzymes such as haem oxygenase-1 (HO-1) at least partially temper hemolysis-related pathophysiologic conditions. However, when the hemolytic insult is severe, the defensive systems may become exhausted or diminished and incapable of preventing pathologic conditions related to hemolysis (78, 81).

In ESOP, there has been an ongoing effort to replace the blood-based perfusates with acellular ones, aiming to eliminate dependence on donor blood and the problems associated with banked blood as well as bypass hemolysis and related problems (82–84). Kappler et al. have shown that during four-hour ESHP in a porcine model, the free hemoglobin in the perfusate increased throughout the perfusion by approximately 0.02 mmol/L per hour (85).

Theoretically, the *ex-situ-*perfused thoracic organs may be more vulnerable to the hemolysis-related redox and inflammatory alterations due to absence of the organs involved in scavenging hemolysis products, and possible diminished antioxidant defence particularly in longer *ex-situ* perfusion times.

### Leukocytes

In general, similar to the other pathologic inflammatory conditions, leukocytes are considered one of the main players in the inflammatory and oxidative stress responses induced during ECLS.

During application of ECLS, neutrophils and monocytes are activated mainly as a result of activation of complement system. Several other factors, including the contact system, thrombin, histamine, heparin, cytokines, neutrophil-activating peptide 2 released from activated platelets, and interactions with platelets, also contribute to the activation of neutrophils. In addition, thrombin triggers monocyte activation both directly (through thrombin receptors on monocytes) and indirectly, (by triggering formation of platelet-monocyte conjugation, similar to the complement system) (86, 87). Activated neutrophils release the contents of their granules, including lysozymes, myeloperoxidase, elastase, hydrogen peroxide, and reactive oxygen and nitrogen species (RONS) (6, 53, 64, 73). Similarly, during ECLS, activated monocytes (also increased in number) produce and release various pro-inflammatory cytokines including TNF-α, and IL-1β, and prostaglandins mainly with inflammatory effects such as prostaglandin (PG)-F2α and PG-E2 (88–90). While non-classical monocytes induce oxidative stress in the vascular system and cause endothelial dysfunction, intermediate monocytes may be involved in induction of the systemic inflammatory responses related to ECC (91). Paradoxically, some monocytes express haemoglobin scavenger receptor (CD163) and may exert anti-inflammatory effects by removal of the potent pro-oxidant haemoglobin-haptoglobin complexes formed due to hemolysis during ECC (92).

In clinical ECLS studies, observations regarding lymphocytic sub-populations have been conflicting. While some studies have reported no change in either the number of lymphocytes, or their activation during application of ECLS (6, 93, 94), others report evidence of an initial increase in the absolute number T-cells, natural killer cells, and suppressor T-cells during ECLS, followed by a reduction to lower than normal values in the days after ECLS is discontinued (95). Evidence on the alterations in other subpopulations of leukocytes during ESOP is currently lacking.

### Endothelial Cells

The endothelium is an active player in different physiologic functions, including controlling vascular tone and permeability, hemostasis, and immune system responses. Conditions that are related to inflammation and oxidative stress (such as ECC), lead to stimulation and activation of the endothelial cells similar to SIRS (5, 96).

Stimulated by various factors including anaphylatoxins, thrombin, and pro-inflammatory cytokines (most potently TNF-α and IL-1β) during ECLS, the expression of P-selectin and E-selectin on the surface of endothelial cells, and L-selectin of neutrophils increase, mediating the low-affinity, reversible rolling of the neutrophils along the vascular endothelium. Further interactions between the endothelial cells and leukocytes mediates transmigration of leukocytes into the extravascular compartment and exacerbation of the inflammatory responses (6, 93). On the other hand, the neutrophil elastase introduced into the endothelial cells leads to generation of superoxide anion by xanthine oxidase. Production of superoxide anion in turn reduces the intracellular ferritin-bound ferric iron (Fe^3+^) to unstable ferrous iron (Fe^2+^) that will cause/exacerbate oxidative stress through the Fenton reaction. The production of NO by nitric oxide synthase (NOS), which decreases the adhesion between neutrophil and endothelial cells, and scavenges superoxide to create peroxynitrite anion, partially compensates for this. However, in high concentrations or longer exposure times, peroxynitrite may directly incorporate a nitro group (−NO_2_) to various tyrosine (Tyr) residues of different proteins/enzymes. Some of these residues include the tyrosines located near charged amino acids or on a loop structure, Tyr34 in manganese superoxide dismutase (MnSOD) and Tyr430 in prostacyclin synthase. This Incorporation can inhibit/alter their enzymatic function and structure and negatively affect normal cellular processes (97–99). The circulating pro-inflammatory cytokines can also directly stimulate endothelial cells leading to a pathologic increase in permeability causing tissue edema and impaired oxygen exchange causing multiple organ dysfunction (4, 5, 97).

Endothelial damage is accepted as an important phenomenon occurring during ESLP, that can decrease the quality of the potential lung graft. The limited data have reported induction of various markers of endothelial activation and damage, including intracellular adhesion molecule-1 (ICAM-1), vascular cell adhesion molecule-1 (VCAM-1), syndecan-1, hyaluronan, and heparan sulphate in the perfusate during

ESLP of human lungs, which had a negative correlation with the outcomes of preservation and transplantation (100–102). In a recent experimental ESHP study of healthy porcine hearts by our team, we demonstrated that the perfusate soluble ICAM-1 and VCAM-1 significantly increased over time alongside different pro-inflammatory cytokines, while the cardiac function and vascular tone (measured by increased coronary flow) decreased, and myocardial tissue developed significant edema (19). These observations strongly suggest an interaction between ECC, inflammation, activation and damage of the coronary endothelial cells, and the functional decline of the *ex-situ*-perfused organs.

## Cytokines

Activation of the innate immune system by pathogens or cellular stressors/stimuli or tissue damage results in the production of pro-inflammatory cytokines that may be accompanied by anti-inflammatory cytokines including IL-10 (66). Cytokines are small-sized proteins with an integral role in immune system responses. Besides, they have various functions in the cell including signaling (5, 103). Generated in clusters, cytokines not only stimulate other cells (immune and non-immune) and alter their function and/or structural integrity, but also induce the production of more cytokines leading to aggravated concentrations of circulating cytokines known as “cytokine storm” (5, 66, 104). The severely induced immune system and high cytokine concentrations cause cellular damage, compromises organ function, and may lead to multiple organ dysfunction syndrome and death (66, 105). As discussed earlier in the manuscript, and demonstrated in the **Supplementary Table 1**, various cytokines have been reported to increase in the setting of ECLS and ESOP. Evidence suggests that the high level of cytokines induced in ECLS is associated with poor short-term and long-term patient outcomes (105–107). Despite the known effects of cytokines in further induction of the immune system and inflammatory and oxidative processes, the effects of cytokine removal during ECLS have been unclear. While application of the technique of cytokine removal with hemoadsorption (CytoSorb device) has been reported to be safe, its beneficial effects on cytokine removal and patient outcomes have been debatable in both CPB and ECMO studies. In a pilot study, Poli et al. reported that using the CytoSorb device during CPB did not decrease pro-inflammatory or anti-inflammatory cytokines, and did not improve clinical outcomes (108). Similarly, Bernardi et al. reported that application of the CytoSorb during CPB in patients undergoing cardiac surgery was not associated with a decrease in the level of pro-inflammatory cytokine IL-6, catecholamine requirements, or 30-day mortality (107). Stupica et al. showed that corticosteroid treatment during cardiac surgery with CPB led to significantly lower circulating levels of IL-6, IL-8 and TNF-αcompared to CytoSorb-applied group, and naïve CPB control group. Yet, the reduction of cytokine values did not improve clinical outcomes, including cardiac function, in any of the groups (109). However, Nemeth et al. showed that while cytokine adsorption during cardiac transplantation surgery and CPB did not diminish the inflammatory responses but was associated with a tendency to improve patient outcomes such as shorter mechanical ventilation and hospital and ICU stay, as well as lower vasopressor requirements (110).

In a case report by Bruengar et al. ECMO support in a patient with cardiogenic sepsis shock, hemoadsorption with CytoSorb successfully removed IL-6 along some other inflammatory molecules such as C-reactive protein, and lead to lower than the usual need for vasopressor agents (111). However, Lother et al. showed that incorporation of cytokine adsorber into the ECMO circuit did not change the vasopressor or inotrope requirements in critically ill patients (112).

It must be remembered that in the ECLS setting the baseline cytokine levels, duration of ECLS, duration of application of hemoadsorption, as well as genetics and original condition and diseases of the patients affect the levels of cytokines (103, 113). Additionally ECMO is mostly applied in critically ill patients with an ongoing inflammatory state related to the underlying diagnosis, thus, the effects of cytokine removal during ECMO may not be attributable to attenuation of the circuit-related cytokine production, but rather the original inflammatory responses related to the illness.

In contrast to ECLS, much less is known about the effects of circulating cytokines produced during ESOP on the outcomes of organ perfusion and eventually transplantation as only a few preclinical studies have made conflicting observations. Kakishita et al. showed that removal of cytokines from the perfusate with an absorbent membrane during ESLP of porcine lungs did not improve pulmonary functional status or edema formation (8). On the other hand, in a pig model Iskender et al. showed that cytokine removal by an absorbent device (CytoSorb) during 12 hours ESLP reduced lung tissue injury and edema, tissue neutrophil infiltration and improved compliance (18). To our knowledge there are currently no studies investigating the effects of active cytokine removal with techniques such as CytoSorb during ESHP. However, Sanda et al. showed that reduced concentrations of circulating cytokines with adding methylprednisolon to the perfusate in a DCD pig model of ESHP did not improve the functional status of the heart during ESHP (61).

Theoretically, circulating cytokines during ESOP not only may directly damage the donated organs and negatively affect the outcomes of transplantation, but also can make it difficult to assess the viability of the perfused organ in case of a cytokine-related reversible functional decline. Moreover, in any of the discussed settings, it is almost impossible to support a single type of cytokine as the one responsible for inflammation-related effects. Moreover, it must be taken into account that despite being an important force in inflammatory and oxidative stress responses, cytokine production is merely one aspect of the highly sophisticated, orchestrated immune and stress responses (5, 103). Thus, robust preclinical, clinical, and transplantation studies are necessary to evaluate the direct effects of circulating cytokines during ECLS and ESOP and the potential benefits of focused cytokine removal.

## Hyperoxia

Hyperoxia is considered as one of the main reasons behind generation of ROS and oxidative stress during ECLS, and occurs routinely during CPB as a result of the aimed supra-physiological partial pressure of oxygen (PaO_2_) in an effort to prevent hypoxia (67, 114). The oxidative stress caused by hyperoxia serves to further exacerbate inflammatory responses seen during CPB (114). Clinical studies on pediatric patients have suggested that hyperoxic CPB (PaO_2_: 150 to180-200 mmHg) -related induction of pro-inflammatory cytokines, markers of oxidative stress (such as 8-isoprostane) and markers of tissue injury (i.e., DAMPS, such as protein S100) may be more severe in cyanotic patients (e.g., single ventricle pediatric patients). However, while the plasma pro-inflammatory cytokines may not be different between hyperoxic and normoxic CPB in non-cyanotic patients, hyperoxic CPB may still cause higher oxidative stress and tissue damage (115, 116).

In general, a considerable number of the patients undergoing ECMO are hypoxic/hypoxemic before the application of ECMO (e.g.' as a result of acute respiratory distress syndrome) thus, the hyperoxia during ECMO may be lead to an exaggerated production of RONS (117). Also, hyperoxia is a more prominent condition in venovenous ECMO (VV-ECMO) where the hyperoxic blood returns to the venous system, directly perfusing the damaged pulmonary vessels (21). Experimentally, a positive correlation has been observed between the tissue expression of markers of oxidative stress, with PaO_2_ during ECMO (118). Also, higher expression of pro-inflammatory cytokines and pulmonary tissue edema, and lower expression of the anti-inflammatory IL-10 in PaO_2_ ≥300 mmHg compared to PaO_2_ 100-299 mmHg during 120 minutes of ECMO has been observed (119). Clinically, it has been suggested that PaO_2_ ≥193 mmHg during the first 48 hour of ECMO may be associated with higher risk of mortality, and some adverse outcomes, such as need for renal dialysis (120, 121).

The optimal PaO_2_ is even more uncertain in ESHP. During the 20^th^ century, ESHP was conduced with blood-free perfusates that would maintain a pH of 7.40 using 95% oxygen flow and PaO_2_≥450 mmHg that was much higher than physiologic values (PaO_2_:75-100 mmHg). Later, with addition of RBC to the perfusate to reach a hematocrit of 35%, the oxygen delivery of the perfusate to the heart improved with more physiologic values of PaO_2_ (122). More recently, in the experimental studies using a RBC-containing perfusate (either whole blood or separated RBCs), the PaO_2_ is maintained mostly in the range of 100≤PaO2<200 mmHg, which is able to provide a physiological oxygen saturation (20, 123, 124). Interestingly, in ESLP, while most common approach in ESLP has been using a deoxygenated perfusate containing 6% oxygen, In a rat model of ESLP and lung transplantation, Noda et al. demonstrated that the perfusate containing 40% or 60% oxygen, when compared to 6%, or 100%, showed significantly lower inflammation expression of the inflammatory cytokine genes in the lung tissue during ESLP, while the group with 40% oxygen in perfusate showed the best post-transplantation pulmonary function (125). These results suggest that potentially, while hyperoxia may simply refer to administration of oxygen in percentages higher than room air (FIO_2_>21%) it is yet to be well defined as it has been described it in a wide range by different studies (100>PaO_2_≥485 mmHg). More over, the efficient and safe oxygenation values may highly depend on the setting, pre-existing conditions, and in the case of ESOP, depending on the organ’s specific needs, thus more studies are warranted to define the safe and efficient oxygenation values in different types of ECC.

## Ischemia-Reperfusion Injury

The pathophysiology of the IRI has been well described before (126, 127). With cross-clamping the aorta and application of cardioplegic solution during cardiac surgery involving CPB, the heart undergoes global ischemia. Also, not only do the lungs become partially ischemic as a result of loss of flow through the bronchial arteries, the peripheral organs may be subjected to global hypoperfusion. The abrupt reperfusion with removal of the cross-clamp leads to IRI, affecting the patients outcomes negatively (6, 128).

The application of ECMO is not necessarily associated with IRI. Though, given the indications of ECMO (respiratory and/or cardiac failure), the organs may have been subjected to different degrees of ischemia/hypoxia and hypoperfusion, thus the hyperoxic perfusion *via* ECMO leads to massive production of RONS that contribute significantly to exacerbation of oxidative stress and inflammatory responses (67). Electron spin resonance spectroscopy evaluation has suggested that production of the reactive oxygen occurs throughout the application of ECLS, going beyond the RONS production in the context of IRI (129).

In ESOP, when compared to standard donor organ preservation method (i.e., SCS), the duration of cold ischemia is considerably shorter. However, ischemia still occurs with the hypoperfusion period following the withdrawal of the life support in the donor, during procurement of the organ, and during mounting of the organ on the *ex-situ* perfusion apparatus (20). More over, in case of DCD, longer warm ischemic times are expected due to the mandatory “no touch” period that is required to confirm the irreversible loss of circulatory function before organ procurement proceeds. Thus, the *ex-situ*-perfused organs are naturally affected by IRI during early perfusion and may also further trigger inflammatory responses and tissue damage that will follow during *ex-situ* perfusion (130, 131). IRI in the setting of ESOP is a contributor to ROS during early perfusion.

## Absorption of Antioxidants/Decreased Antioxidative Potential

Growing evidence suggest that the bioavailability of micronutrient and trace elements such as copper, zinc, selenium, manganese, necessary for the function of the antioxidative enzymes, as well as the non-enzymatic antioxidants such as, uric acid, vitamins C and E, decrease during ECC (132–134). The decreased values of micronutrients/trace elements during ECC may be related to acute-phase redistribution as a result of the increased inflammatory response, absorption to the ECC circuit, and excretion in bodily fluids/perfusate (21, 67, 133). Such a decline can lead to exacerbation of inflammatory responses and oxidative stress and related damage to the exposed tissues/organs. The status of micronutrients and trace elements during ESOP and its association with inflammation and oxidative stress and outcomes of ex-situ organ preservation and transplantation has been almost unexplored so far. In a porcine model of extended ESLP for 24 hours, Buchko et al. showed that continuous supplementation of the perfusate with the total parenteral solutions that also contain micronutrients, lead to lower inflammation, and improved graft function compared to the controls, however, the specific effects of the micronutrients/trace elements had not been explored (135). Also, to our best knowledge, there is no data available on the potential alterations in the levels of micronutrients and trace elements during ESHP, and implications of their decline, or supplementation on the preservation of the donated heart during ESHP, or the outcomes of transplantation.

## Clinical Significance of Inflammation and Oxidative Stress

Despite the induction of inflammatory responses and oxidative stress during ECLS being reported frequently, the clinical significance of these findings has been a matter of debate, since the existing data is very limited, and is mostly related to preclinical studies. Among a vast range of the markers of inflammation and oxidative stress reported to increase during or after ECLS, IL-6, IL-8 induced during CPB have been associated with higher incidence of post operative cardiac dysfunction, infection, and longer ICU and hospital length of stay, with Il-6 specifically being associated with pulmonary dysfunction after CPB. Similarly, increased levels of IL-6, IL-8, and TNF-α are reported to be associated with higher in hospital mortality in patients undergoing ECMO, particularly VV-ECMO (4).

In the ESOP setting, it has been demonstrated that lower induction of the markers of inflammation and/or oxidative stress in tissue and/or the perfusate during *ex-situ* perfusion is associated with better preservation of functional status of the perfused heart or lung in preclinical studies (56, 136). Andreasson et al. have reported that lower levels of inflammatory cytokines in the perfusate and BAL in ESLP of a group of human donor lungs initially deemed non-transplantable, was associated with better recovery during ESLP and subsequent successful transplantation. Their findings suggest a predictive value for combined perfusate IL-8 and IL-1β during ESLP for prediction of the early outcomes of lung transplantation (137). In a single center study by Sage et al. a scoring system based on the perfusate levels of IL-6 and IL-8 can be used to predict the early outcomes of lung transplantation including the incidence of primary graft dysfunction (138). Although these studies provide very useful information about the implications of inflammation and oxidative stress-induced during ESLP on recovery of the lungs during ESLP and the outcomes of transplantation, the results may also be affected by the variables related to the donor’s organ characteristics and recipient factors. In the setting of ESHP, preclinical studies report induction of the markers of oxidative stress and inflammation during ESHP in both perfusate and myocardial tissue even in the negligible damaged animal hearts (as opposed to DCD models) (19, 63, 139), even with a leukocyte-depleted perfusate (140). In a study of ESHP of DCD rat hearts by Lu et al., melatonin attenuated the induction of IL-11β, IL-18, IL-6 and TNF-α as well as the markers of oxidative injury such as malondialdehyde and 4-hydroxynonenal in the myocardial tissue, resulting in superior functional status (141). However, in a DCD porcine model of ESHP, Sandha et al. showed that attenuating of the induction of the inflammatory responses during ESHP, although lead to lower edema formation in the heart, was not associated with improvement of myocardial function during ESHP. This observations may be at least partially related to the warm ischemic insult occurring around the procurement time in DCD, and not exclusively reflecting the effects of ECC. Yet, there is a scarcity of data on the implications of these responses on the outcomes of ESHP and heart transplantation in preclinical studies, and to our best knowledge, there is no clinical data on this issue. More studies, with adjustment to the donor and recipient conditions are warranted to evaluate the clinical significance of these phenomena that occur during ESHP on the outcomes of subsequent heart transplantation.

## Targeting Inflammation and Oxidative Stress in Extracorporeal Circulation

### Circuit Optimization

With the identification of inflammation and oxidative stress in ECLS, attention was turned to 1) determining the components of the circuit that may have a prominent role in this phenomenon, and 2) how to improve the general aspects of the circuits to prevent or minimize these effects.

The surface modification of the circuit, intended to replicate the anti-thrombotic and anti-inflammatory properties of the endothelium, include application of biomimetic surfaces (e.g.' using heparin or direct thrombin inhibitors), biopassive surfaces (e.g.' using phosphorylcholine, or albumin), as well as more experimental attempts at endothelization of circuit components (142–144). Both ECLS and ESOP systems typically make use of polyvinyl chloride (PVC) tubing (with the latter taking the lead from the former).

The most commonly used biomimetic approach, which seeks to replicate the antithrombotic and anti-inflammatory properties to endothelium, is heparin-bonding. Though there has been little direct study of heparin-bonding in ESOP circuitry, it has been shown to reduce cellular activation and release of inflammatory mediators in clinical studies of ECLS, as well as *in vivo* models, and is even associated with improved clinical outcomes, such as decreased ICU stay and lower rates of post-operative atrial fibrillation (145–148). Priming the circuit with a heparin-albumin solution can have a similar effect, given the capacity of PVC to absorb plasma proteins (149). Nitric oxide (NO)-bounded materials have also been used in ECLS as a means to prevent platelet activation and aggregation (150). Though, their direct effect on inflammation has not been well studied, they are known the have inherent bactericidal properties (151).

Biopassive approaches, by contrast, seek to make the circuitry more inert. A common technique is coating with phosphorylcholine, the main phospholipid component of cell membranes. It is thought that their formation of a biomembrane surface leads to reduced thrombin formation, though their effect on inflammation is not well known. Plasticisation of circuit components with the amphiphilic polymer, poly-2-methoxyethylacrylate, has also been shown to reduce platelet adhesion and aggregation in ECLS, as well as decrease complement activation and inflammatory markers (152–154).

Considering the aforementioned, currently the best evidence exists for heparin-coated and third-generation heparin-polymer coated ECLS circuits (commercially-available), which have been shown to improve biocompatibility and attenuate inflammation, in both experimental and clinical studies, by suppressing various pathways involved. They have even been directly linked to improved clinical outcomes, though they are more expensive (142–144).

Application of miniaturized extracorporeal circuits (MECC), with lower surface area compared to conventional circuits (shorter tubing length, eliminated venous reservoir and suction device), being fully closed to prevent air-blood interface (even as a CPB circuit), and lower requirements for priming volume and blood transfusion, has been associated with significantly lower inflammation and oxidative stress. The patient outcomes using MECC circuit have been either similar with the conventional circuit, or have shown some beneficial effects including lower incidence of post-operative atrial fibrillation (AF), myocardial infarction, renal failure, and shorter duration of ICU stay and intubation time. However, they may not be suitable for highly complicated procedures/surgeries (21, 64, 155, 156).

Regardless of the size of the circuit, there is limited data on the relative contribution of each circuit component to initiating inflammation and oxidative stress. It has been suggested that using smaller oxygenators and connectors, smaller pumps needing higher pump speeds, and additional in-line filters are associated with higher shear stress and hemolysis during perfusion, exacerbating oxidative stress and inflammation (67, 157).

There is very limited scientific data on the effects of circuit optimization in ESOP. This may be partially related to the fact that the concept of clinical ESOP with modern technology as a method for preservation and potential recovery of donor heart and lung is still in its infancy. Moreover, there is incredible variability in the custom-built circuits used in experimental studies on ESOP, also affected by the size of the experimental animal model used (3, 158) making it very difficult to evaluate/compare these circuits and their individual components for induction of inflammation/oxidative stress during perfusion. While there are currently four different devices commercially available for clinical normothermic ESLP, there is only one for clinical normothermic ESHP (3, 159). Studies using clinically relevant platforms and circuits are necessary for evaluation of biocompatibility in ESOP setting in terms of reducing inflammation and oxidative stress.

### Leukocyte Filtration

Considering the importance of the role attributed to leukocytes in induction of inflammatory and oxidative stress responses during ECC, eliminating this phenomenon is believed to bear a critical potential for protection of the organs in this setting. Continuous leukocyte filtration with arterial line filters incorporated into the circuit has shown mixed results in terms of attenuation of inflammation, oxidative stress, and organ dysfunction during and after CPB. It has also been reported that placing the leukocyte filter in venous line, while still efficiently filtering the leukocytes, may cause lower leukocyte damage and thus, better attenuate inflammation (160–164).

In critically ill neonates placed on ECMO, transfusion of leukoreduced blood *via* leukocyte filtration is associated with improvement of patient outcomes, including survival (165, 166). On the other hand, prolonged leukocyte filtration, despite continuous reduction of circulating leukocyte count, may increase hemolysis and leukocyte damage, which can induce or exacerbate inflammation and oxidative stress. However, it is difficult to isolate the effects of leukoreduction in ECMO, as they may simply be related to the underlying condition (167).

Leukocyte filters are routinely incorporated into ESLP circuits to attenuate ECC-induced inflammation and oxidative stress (168). However, existing experimental studies have shown contradictory results in terms of attenuating inflammation and improving the outcomes of organ preservation and transplantation (55, 56, 168). Interestingly, our group has also reported similar number of trapped leukocytes in the leukocyte filter between cellular and acellular perfusates, and comparable histological findings between ESLP with or without leukocyte filter (168). These findings may suggest that either most of the cells trapped in the leukocyte filter during ESLP originate from the lung and/or the efficiency of leukocyte filter in ESLP is sub-optimal.

In clinical ESHP, leukoreduced donor blood is routinely used for the perfusate to prime the circuit (169). Leukocyte reduced or depleted perfusate (either using leukocyte filters or centrifuged blood) has been also frequently used in experimental ESHP studies, aiming to reduce inflammation and oxidative stress (57, 123, 170). However, our group has shown that ESHP with either a leukocyte filter or a perfusate containing leukocyte depleted blood is still accompanied with considerable systemic and tissue inflammation and oxidative stress during perfusion without benefiting functional preservation (19, 140).

### Pharmacological Interventions

The optimal pharmacological prevention/attenuation of the inflammation and oxidative stress in the setting of ECC is still a matter of debate. The studies performed to address this phenomena, have applied various agents belonging to the general class of anti-inflammatory medications, as well as the experimental agents. Interestingly while most of these studies are performed in the clinical CPB setting, there a paucity of studies targeting inflammatory and oxidative responses in ECMO, or ESOP. The most widely studied agents in the setting of ECC have been discusses in the following sections.

### Corticosteroids

The prophylactic administration of steroid, with the aim of improving the post-operative patient outcomes in surgeries involving CPB, has shown mixed results in clinical trials **Supplementary Table 2**. Multiple studies have found that corticosteroid-related attenuation of the inflammation and tissue injury and oxidative stress, is associated with improvement of post-operative contractile function of the heart, lower incidence of post-operative atrial fibrillation (AF), decreased requirement for ventilator and circulatory support, and shorter length of hospital and ICU stay without increasing the risk of infections (171–180). Still, some other studies have not detected any beneficial effects for corticosteroid administration in improving post-operative primary and secondary outcomes (173–176, 180). Despite the mixed findings, meta-analytical studies suggests that prophylactic corticosteroids in patients undergoing cardiac surgery involving CPB significantly decreases the length of stay in ICU and hospital and incidence of post-operation AF in both adult and pediatric patient populations, and decreased inflammation has been suggested to be one of the key element in these effects (181–183). The prophylactic steroid administration in this setting is currently recommended by the American Heart Association and the American College of Cardiology (184).

Corticosteroids (mainly methylprednisolone) are also commonly administered in critically ill patients undergoing ECMO, where they are believed to provide benefit through reducing the systemic inflammatory response and compensating for any cortico-adrenal insufficiency. The related clinical trials in general have supported its effect in improving secondary outcomes such as decreasing the need for mechanical ventilation and the length of ICU stay alongside decreasing the inflammatory responses. Moreover, a meta-analysis by Meduri et al. showed that decreased values of inflammatory cytokines in critically ill patients with ECMO who were treated with methylprednisolone for a prolonged period, and early during the course (for more than two weeks, starting in the first two weeks) was also associated with reduced incidence of in-hospital mortality (185, 186)

The addition of corticosteroid (methylprednisolone) to the perfusate during EVOP has been a traditional approach in both experimental and clinical protocols for ESOP (56, 187, 188). The main reason for this intervention has been the attempt to attenuate/prevent the deleterious effect of IRI and related inflammation and oxidative stress that donated organs are subjected to due to ischemia and other stresses occurring during the process of donation and organ procurement. In a rat model of brain death and ESLP, Van Zanden et al. showed that the addition of methylprednisolone to the perfusate led to lower expression of IL-6 and IL-1β genes in the tissue, and lower perfusate IL-6 and improved positive inspiratory pressures compared to controls (189). In a study by Martens et al. administration of methylprednisolone both before the arrest, and during the ESLP in a DCD porcine model led to a significant reduction in IL-1β, IL-8, and TNF-α, together with superior pulmonary compliance in treated lungs compared to the controls. However, the results of this study might have been also affected by pre-arrest administration (preconditioning) of the organs by methylprednisolone, rather than only targeting the inflammatory responses during ESLP (190). Conversely, In the setting of ESHP, the few existing experimental studies have reported little or no beneficial effects for corticosteroids added during ESHP in preventing cardiac functional decline, tissue damage or edema during perfusion (61, 191). However the addition of corticosteroids to the perfusate during ESOP, has been considered as a safe and inexpensive attempt to attenuate the inflammation in the perfused hearts and lungs, regardless of the donation condition and basic status of the donated organ.

### Statins

During the past few decades the anti-inflammatory and antioxidative effects of statins has been recognized (192, 193). Clinical trials and meta-analyses have suggested that preoperative prescription of a statin in patients undergoing cardiac surgery with CPB can attenuate the inflammatory responses and tissue damage induced by extracorporeal circulation, and may improve patient outcomes (4, 193, 194). However, a more recent meta-analysis by An et al. revealed that preoperative administration of statins has the beneficial effect of decreasing the incidence of post-operative AF, but not myocardial infraction or stroke in patients undergoing cardiac surgery requiring CPB (195). Interestingly, the meta-analysis by Putzu et al. suggested an increased risk of acute kidney injury and in hospital mortality with preoperative statins, while the earlier meta-analysis by this group also failed to show any improvement in patient outcomes such as mortality and incidence of post-operative AF (4, 196, 197).

Statins have been also used adjacent to VV ECMO for treatment of respiratory pathologies such as ARDS (186), however the effect of statins on inflammatory and oxidative stress occurring during extracorporeal circulation has not been investigated. Similarly, in ESHP, statins have been investigated to attenuate myocardial IRI for their anti-inflammatory and antioxidant effects, and have shown beneficial effects for this purpose (198, 199), but not specifically for attenuating ECC-related inflammation and oxidative stress in the setting of ESOP.

### Phosphodiesterase Inhibitors

The anti-inflammatory effects of phosphodiesterase inhibitors (PDEIs) in ECLS have been somewhat controversial. Experimental and clinical studies suggest that PDEIs can improve myocardial energetics, and ameliorate inflammation, and may also have protective effects on endothelial and pulmonary tissue as well (200–204).

The PDEIs have been also investigated in EVLP to address conditions such as pulmonary hypertension and IRI, and have shown beneficial effects including vasorelaxation (205–207). However, experimental administration of PDEIs in EVLP of discarded human donor lungs had no effect on inflammation, and did not improve the physiological status of the lungs, or prevent tissue edema (208). Although cardioprotective and therapeutic properties of different families of PDEIs have been evaluated in the setting of ESHP (209, 210), their effect on mitigating the inflammatory responses and/or improving the functional preservation of the *ex-situ*-perfused heart is still unknown.

### Complement Inhibition

Experimental and clinical studies evaluating different complement inhibitors including APT070 and TP10 (C3 and C5 activation inhibitor) and Pexelizumab (a recombinant antibody binding to C5) have reported a significant reduction in active complement proteins, and better protection of the lungs and myocardium during CPB, and improvement in patient outcomes (211–213). However, some of these studies failed to show an association between diminished complement activation and inflammation. It has been shown that heparin-coated ECMO circuits, lead to attenuation in complement activation due to complement-inhibitory effects of heparin (214).

As a technique to study the physiology and pathology of the heart, ESHP has been used to evaluate the effects of complement activation and inhibition on the myocardium (215, 216), but there is a paucity of data on the effect of complement inhibition during ESHP with blood derived perfusates, or during ESLP.

### Serine Protease Inhibitors

Clinical and experimental studies on ECLS suggest that serine protease inhibitors (SPIs) may attenuate the ECC-related inflammation by decreasing the activation of the contact system (through preservation of kalikrein inhibitor activity), coagulation pathway (inhibition of plasmin), complement system (decreased production/activation of complement factors), and by modulating leukocyte activation, which may lead to organ-protective effects during ECC **Supplementary Table 2** (184, 217–220). Though, there are still some conflicting reports in the literature, such as the observational study by Mangano et al., which reported significantly higher incidence of adverse cardiac, cerebral, and renal events in patients receiving aprotinin (184, 221).

There is evidence from *ex-situ* perfusion of animal and discarded human lungs that SPIs may decrease systemic and tissue inflammation, improve *ex-situ* protection of the lungs, and attenuate tissue edema (222, 223). The anti-inflammatory and tissue protecting effects of SPIs has been assessed in the context of ESHP to protect the myocardium from IRI, and have shown cardioprotective effects against it (224, 225), but not in attenuation of the inflammatory and oxidative stress responses related to ECC specifically.

### Antioxidants

Administration of different antioxidants either before, or during ECLS may lower production of RONS, attenuate oxidative stress, inflammation, and related tissue damage, and improve heart and lung protection and function, and patient outcomes (226–230). Although some studies could not detect any improvement in the pro-inflammatory profile, organ function, or patient outcomes with administration of some well known antioxidants, such as vitamins and coenzyme Q10 in the context of CPB application (231–233), a meta-analysis of the clinical trials revealed that application of NAC, polyunsaturated fatty acids (PUFA), and vitamin C in surgeries applying CPB is associated with lower risk of post-operative AF, and improved patient outcomes (mortality in NAC, and length of hospital stay in PUFA) (234). Similarly, a recent meta-analysis reported that perioperative administration of vitamin C is associated with lower incidence of post-operative arrhythmia, shorter mechanical ventilation period and shorter length of hospital and ICU stay, though not improved mortality (235).

There is a significant paucity of data on the effects of antioxidants in ECMO treatment, despite the fact that the endogenous antioxidant defence system is impaired in the critically ill patients requiring ECMO (67). Experimental and clinical studies are needed to address the effects of antioxidants administered during ECMO treatment on systemic inflammation and oxidative stress, organ damage, and patient outcomes.

Experimental administration of antioxidative agents either in the perfusate, or *via* inhalation during ESLP can decreased generation of RONS, oxidative stress-related protein modifications, inflammation, improve lung tissue protection and attenuate tissue edema, and improve post-transplantation respiratory function of the lung (236–239). Most of these ESLP studies have been performed in discarded lungs that had endured significant ischemia thus, severe IRI would be expected upon reperfusion with ESLP, which may mask the effects of antioxidants on extracorporeal related inflammation and oxidative stress. However, in a rat model of minimally damaged donor lungs (which has not endured clinically relevant ischemia), administration of the antioxidant 2% hydrogen through inhalation was reported to decrease expression of IL-6, IL-1β, and TNF-α mRNA compared to controls, and to improve metabolic profile of the lungs and post-transplantation pulmonary function (240). The results of this study not only support the detrimental effects of ECC-related oxidative stress and inflammation on preservation of the lung during ESLP, and on outcomes of transplantation, but also support the beneficial effects of antioxidative support for the lungs during ESLP, regardless of the perioperative insults.

With increasing interest in the use of ESHP to facilitate the transplantation of DCD hearts, IRI has been a focus of research in this field. Animal models of *ex-situ* perfusion of the hearts subjected to long ischemic times have reported that administration of antioxidants in ESHP reduces oxidative stress and related modifications, inflammation, and myocardial edema, and may be associated with better preservation of coronary endothelium, myocardial tissue integrity, and organelles such as mitochondria in cardiac tissue (241–244). While these studies are focused on attenuating the myocardial IRI, to our knowledge there are no available studies on the cardioprotective effects of antioxidants against the ECC-related oxidative stress during ESHP.

## Conclusion

Inflammation and oxidative stress are induced during ECLS, as well as ESOP. While these phenomena have been explored in more details in ECLS, many aspects remain obscure in ESOP studies. Similarly, the implications of these reactions are not yet fully understood, and there is a considerable scarcity of information on the effect of ongoing inflammation and oxidative stress in ESOP on functional viability of the *ex-situ* perfused hearts and lungs, and on the outcomes of transplantation in these organs. There has been a significant variability in both experimental and clinical study designs and the anti-inflammatory and antioxidative interventions applied in the ECLS setting, as well as the existing limited studies in ESOP. The variability of the custom-built circuits in experimental extracorporeal perfusion studies may also contribute to some of the controversial findings in these studies. Increased knowledge of the ECC-related inflammation and oxidative stress and their outcomes, particularly in ESOP, and targeting those with clinically-translatable methods will help in 1) providing a safer life support for the critically ill patients or the patients undergoing cardiac surgery;, and 2) improving ESHP protocols to offer optimal organ preservation and potentially improvement of the graft condition.

## Author Contributions

All the authors contributed to the content of this manuscript. SH and JH have performed the literature review and summarizing, as well as categorizing the studies and sections and preparation of the study tables. SH has written the manuscript. DF is the corresponding author and has been involved with the selection of the topic, and has reviewed and guided the preparation of the manuscript. All authors contributed to the article and approved the submitted version.

## Conflict of Interest

The authors declare that the research was conducted in the absence of any commercial or financial relationships that could be construed as a potential conflict of interest.

## Publisher’s Note

All claims expressed in this article are solely those of the authors and do not necessarily represent those of their affiliated organizations, or those of the publisher, the editors and the reviewers. Any product that may be evaluated in this article, or claim that may be made by its manufacturer, is not guaranteed or endorsed by the publisher.

## Supplementary Material

Supplementary Table 1 Highlighted studies on inflammatory and oxidative stress responses induced during extracorporeal life support, and *ex-situ* thoracic organ perfusion. BAL, bronchoalveolar lavage; Bcl-2, B-cell lymphoma-2; CABG, coronary artery bypass grafting; CD40, cluster of differentiation 40; Clin, clinical study; CPB, cardiopulmonary bypass; C3a, cleaved complement component C3 fragment a; C4a, cleaved complement component C4 fragment a; C5b9, cleaved complement component C5 fragment b9; ECMO, extracorporeal membrane oxygenation; Exp, experimental study; DBD, donation after brainstem death; DCD, donation after circulatory death; ESHP, *ex-situ* heart perfusion; ESLP, *ex-situ* lung perfusion; HO-1, hemoxygenase-1; IL-, interleukin; MDA, malondialdehyde; MCP-1, monocyte chemoattractant protein-1; MIF, macrophage migration inhibitory factor; MMp-2, matrix metalloproteinase-2; NADPH, Reduced nicotinamide adenine dinucleotide phosphate; PGF2, Prostaglandin F2; PMN, polymorphonuclear; SIRS, systemic inflammatory response syndrome; TNF-α, tumor necrosis factor α; TXA, tranexamic acid; VCAM-1, vascular cell adhesion molecule-1; VA-, venoarterial; VV-, venovenous

Supplementary Table 2 Pharmacologic interventions to reduce oxidative stress & inflammation in extracorporeal circulation. C3a, cleaved complement component C3 fragment a; CD11b, cluster of differentiation 11b; CK, creatine kinase; CK-MB, MB isoenzyme of creatine kinase; CPB, cardiopulmonary bypass; ECMO, extracorporeal membrane oxygenation; ESHP, *ex-situ* heart perfusion; ESLP, *ex-situ* lung perfusion; ICU, intensive care unit; IL-, interleukin;LDH, lactate dehydrogenase; MIP-1, macrophage inflammatory protein-1, MMP, matrix metalloproteinase-2; MPO, myeloperoxidase; NADPH, Reduced nicotinamide adenine dinucleotide phosphate; PAR, poly(adenosine diphosphate-ribose); PGF2, Prostaglandin F2; PMN, polymorphonuclear; POD, post-operative day; RONS, reactive oxygen and nitrogen species; SIRS, systemic inflammatory response syndrome; TNF-α, tumor necrosis factor α; VCAM-1, vascular cell adhesion molecule-1; VA-, venoarterial

## References

[1] McRae K de Perrot M. Principles and Indications of Extracorporeal Life Support in General Thoracic Surgery. J Thorac Dis (2018) 10(Suppl 8):S931–s46. doi: 10.21037/jtd.2018.03.116 PMC5934120 29744220

[2] Griffiths C Scott WE III Ali S Fisher AJ. Maximizing Organs for Donation: The Potential for *Ex Situ* Normothermic Machine Perfusion. QJM: Int J Med (2020). doi: 10.1093/qjmed/hcz321 31943119

[3] Hatami S Freed DH. Machine Perfusion of Donor Heart: State of the Art. Curr Transplant Rep (2019) 6(3):242–50. doi: 10.1007/s40472-019-00251-4

[4] Al-Fares A Pettenuzzo T Del Sorbo L. Extracorporeal Life Support and Systemic Inflammation. Intensive Care Medicine Experimental (ICMx) (2019) 7(Suppl 1):46. doi: 10.1186/s40635-019-0249-y 31346840 PMC6658641

[5] Millar JE Fanning JP McDonald CI McAuley DF Fraser JF. The Inflammatory Response to Extracorporeal Membrane Oxygenation (ECMO): A Review of the Pathophysiology. Crit Care (London England) (2016) 20(1):387. doi: 10.1186/s13054-016-1570-4 PMC5125043 27890016

[6] Warren OJ Smith AJ Alexiou C Rogers PL Jawad N Vincent C. The Inflammatory Response to Cardiopulmonary Bypass: Part 1–Mechanisms of Pathogenesis. J Cardiothorac Vasc Anesth (2009) 23(2):223–31. doi: 10.1053/j.jvca.2008.08.007 18930659

[7] Hatami S White C Shan S Haromy A Qi X Ondrus M. Myocardial Functional Decline During Prolonged *Ex Situ* Heart Perfusion. Ann Thorac Surg (2019) 108(2):499–507. doi: 10.1016/j.athoracsur.2019.01.076 30872100

[8] Kakishita T Oto T Hori S Miyoshi K Otani S Yamamoto S. Suppression of Inflammatory Cytokines During *Ex Vivo* Lung Perfusion With an Adsorbent Membrane. Ann Thorac Surg (2010) 89(6):1773–9. doi: 10.1016/j.athoracsur.2010.02.077 20494026

[9] Boettcher W Merkle F Weitkemper HH. History of Extracorporeal Circulation: The Conceptional and Developmental Period. J Extra-corporeal Technol (2003) 35(3):172–83. 14653416

[10] Passaroni AC Silva MA Yoshida WB. Cardiopulmonary Bypass: Development of John Gibbon's Heart-Lung Machine. Rev Bras Cir Cardiovasc Orgao Oficial Soc Bras Cir Cardiovasc (2015) 30(2):235–45. doi: 10.5935/1678-9741.20150021 PMC4462970 26107456

[11] Lim MW. The History of Extracorporeal Oxygenators*. Anaesthesia (2006) 61(10):984–95. doi: 10.1111/j.1365-2044.2006.04781.x 16978315

[12] Hessel EA 2nd. A Brief History of Cardiopulmonary Bypass. Semin Cardiothorac Vasc Anesth (2014) 18(2):87–100. doi: 10.1177/1089253214530045 24728884

[13] Mosier JM Kelsey M Raz Y Gunnerson KJ Meyer R Hypes CD. Extracorporeal Membrane Oxygenation (ECMO) for Critically Ill Adults in the Emergency Department: History, Current Applications, and Future Directions. Crit Care (London England) (2015) 19:431. doi: 10.1186/s13054-015-1155-7 PMC4699333 26672979

[14] Featherstone PJ Ball CM. The Early History of Extracorporeal Membrane Oxygenation. Anesth Intensive Care (2018) 46(6):555–7. doi: 10.1177/0310057X1804600601 30447660

[15] Liao R Podesser BK Lim CC. The Continuing Evolution of the Langendorff and Ejecting Murine Heart: New Advances in Cardiac Phenotyping. Am J Physiol Heart Circulatory Physiol (2012) 303(2):H156–67. doi: 10.1152/ajpheart.00333.2012 PMC3404701 22636675

[16] Wang L MacGowan GA Ali S Dark JH. *Ex Situ* Heart Perfusion: The Past, the Present, and the Future. J Heart Lung Transplant Off Publ Int Soc Heart Transplant (2020) 40:69–86. doi: 10.1016/j.healun.2020.10.004 33162304

[17] Hosgood SA Moore T Kleverlaan T Adams T Nicholson ML. Haemoadsorption Reduces the Inflammatory Response and Improves Blood Flow During *Ex Vivo* Renal Perfusion in an Experimental Model. J Transl Med (2017) 15(1):216. doi: 10.1186/s12967-017-1314-5 29070045 PMC5657103

[18] Iskender I Cosgun T Arni S Trinkwitz M Fehlings S Yamada Y. Cytokine Filtration Modulates Pulmonary Metabolism and Edema Formation During *Ex Vivo* Lung Perfusion. J Heart Lung Transplant Off Publ Int Soc Heart Transplant (2017) 37:283–91. doi: 10.1016/j.healun.2017.05.021 28587802

[19] Hatami S White C Qi X Buchko M Ondrus M Kinnear A. Immunity and Stress Responses Are Induced During *Ex-Situ* Heart Perfusion. Circ Heart Fail (2020) 13(6):e006552. doi: 10.1161/CIRCHEARTFAILURE.119.006552 32498623

[20] Hatami S White CW Ondrus M Qi X Buchko M Himmat S. Normothermic *Ex Situ* Heart Perfusion in Working Mode: Assessment of Cardiac Function and Metabolism. J Vis Exp JoVE (2019) 143). doi: 10.3791/58430 30688296

[21] McDonald CI Fraser JF Coombes JS Fung YL. Oxidative Stress During Extracorporeal Circulation. Eur J Cardio-Thorac Surg Off J Eur Assoc Cardio-Thorac Surg (2014) 46(6):937–43. doi: 10.1093/ejcts/ezt637 24482384

[22] Haeffner-Cavaillon N Roussellier N Ponzio O Carreno MP Laude M Carpentier A. Induction of Interleukin-1 Production in Patients Undergoing Cardiopulmonary Bypass. J Thorac Cardiovasc Surg (1989) 98(6):1100–6. doi: 10.1016/S0022-5223(19)34325-9 2586127

[23] Steinberg JB Kapelanski DP Olson JD Weiler JM. Cytokine and Complement Levels in Patients Undergoing Cardiopulmonary Bypass. J Thorac Cardiovasc Surg (1993) 106(6):1008–16. doi: 10.1016/S0022-5223(19)33971-6 8246532

[24] Hirthler M Simoni J Dickson M. Elevated Levels of Endotoxin, Oxygen-Derived Free Radicals, and Cytokines During Extracorporeal Membrane Oxygenation. J Pediatr Surg (1992) 27(9):1199–202. doi: 10.1016/0022-3468(92)90787-8 1432529

[25] Ohata T Sawa Y Kadoba K Taniguchi K Ichikawa H Masai T. Normothermia Has Beneficial Effects in Cardiopulmonary Bypass Attenuating Inflammatory Reactions. ASAIO J (American Soc Artif Internal Organs 1992) (1995) 41(3):M288–91. doi: 10.1097/00002480-199507000-00014 8573808

[26] McBride WT Armstrong MA Gilliland H McMurray TJ. The Balance of Pro and Anti-Inflammatory Cytokines in Plasma and Bronchoalveolar Lavage (BAL) at Paediatric Cardiac Surgery. Cytokine (1996) 8(9):724–9. doi: 10.1006/cyto.1996.0096 8932984

[27] Ito H Hamano K Gohra H Katoh T Fujimura Y Tsuboi H. Relationship Between Respiratory Distress and Cytokine Response After Cardiopulmonary Bypass. Surg Today (1997) 27(3):220–5. doi: 10.1007/BF00941649 9068102

[28] Matata BM Galiñanes M. Cardiopulmonary Bypass Exacerbates Oxidative Stress But Does Not Increase Proinflammatory Cytokine Release in Patients With Diabetes Compared With Patients Without Diabetes: Regulatory Effects of Exogenous Nitric Oxide. J Thorac Cardiovasc Surg (2000) 120(1):1–11. doi: 10.1067/mtc.2000.106835 10884648

[29] Golej J Winter P Schoffmann G Kahlbacher H Stoll E Boigner H. Impact of Extracorporeal Membrane Oxygenation Modality on Cytokine Release During Rescue From Infant Hypoxia. Shock (Augusta Ga) (2003) 20(2):110–5. doi: 10.1097/01.shk.0000075571.93053.2c 12865653

[30] Ulus AT Aksoyek A Ozkan M Katircioglu SF Basu S. Cardiopulmonary Bypass as a Cause of Free Radical-Induced Oxidative Stress and Enhanced Blood-Borne Isoprostanes in Humans. Free Radical Biol Med (2003) 34(7):911–7. doi: 10.1016/S0891-5849(03)00030-3 12654480

[31] Zimmermann AK Simon P Seeburger J Hoffmann J Ziemer G Aebert H. Cytokine Gene Expression in Monocytes of Patients Undergoing Cardiopulmonary Bypass Surgery Evaluated by Real-Time PCR. J Cell Mol Med (2003) 7(2):146–56. doi: 10.1111/j.1582-4934.2003.tb00213.x PMC6740292 12927053

[32] Chen YF Tsai WC Lin CC Tsai LY Lee CS Huang CH. Effect of Leukocyte Depletion on Endothelial Cell Activation and Transendothelial Migration of Leukocytes During Cardiopulmonary Bypass. Ann Thorac Surg (2004) 78(2):634–42; discussion 42-3. doi: 10.1016/j.athoracsur.2004.02.091 15276536

[33] Liu JH Shen JM Li L Chang YT. Effects of Fentanyl on Cytokines and MDA During Cardiopulmonary Bypass in Patients Undergoing Valve Replacement. Zhong Nan Xue Xue Bao Yi Xue Ban = J Cent South Univ Med Sci (2005) 30(1):80–3. 15871195

[34] Christen S Finckh B Lykkesfeldt J Gessler P Frese-Schaper M Nielsen P. Oxidative Stress Precedes Peak Systemic Inflammatory Response in Pediatric Patients Undergoing Cardiopulmonary Bypass Operation. Free Radical Biol Med (2005) 38(10):1323–32. doi: 10.1016/j.freeradbiomed.2005.01.016 15855050

[35] de Mendonça-Filho HTF Pereira KC Fontes M Vieira D de Mendonça MLAF Campos L. Circulating Inflammatory Mediators and Organ Dysfunction After Cardiovascular Surgery With Cardiopulmonary Bypass: A Prospective Observational Study. Crit Care (2006) 10(2):R46. doi: 10.1186/cc4857 16542504 PMC1550915

[36] Mildner RJ Taub N Vyas JR Killer HM Firmin RK Field DJ. Cytokine Imbalance in Infants Receiving Extracorporeal Membrane Oxygenation for Respiratory Failure. Neonatology (2005) 88(4):321–7. doi: 10.1159/000087630 16113527

[37] Halter J Steinberg J Fink G Lutz C Picone A Maybury R. Evidence of Systemic Cytokine Release in Patients Undergoing Cardiopulmonary Bypass. J Extra-corporeal Technol (2005) 37(3):272–7. PMC4680784 16350379

[38] Amoureux S Sicard P Korandji C Borey A Benkhadra S Sequeira-Le Grand A. Increase in Levels of BDNF is Associated With Inflammation and Oxidative Stress During Cardiopulmonary Bypass. Int J Biomed Sci IJBS (2008) 4(3):204–11. PMC3614700 23675091

[39] Faragó N Kocsis GF Fehér LZ Csont T Hackler L Jr. Varga-Orvos Z. Gene and Protein Expression Changes in Response to Normoxic Perfusion in Mouse Hearts. J Pharmacol Toxicol Methods (2008) 57(2):145–54. doi: 10.1016/j.vascn.2008.01.001 18304839

[40] McILwain RB Timpa JG Kurundkar AR Holt DW Kelly DR Hartman YE. Plasma Concentrations of Inflammatory Cytokines Rise Rapidly During ECMO-Related SIRS Due to the Release of Preformed Stores in the Intestine. Lab Invest J Tech Methods Pathol (2010) 90(1):128–39. doi: 10.1038/labinvest.2009.119 PMC2799549 19901912

[41] Sadaria MR Smith PD Fullerton DA Justison GA Lee JH Puskas F. Cytokine Expression Profile in Human Lungs Undergoing Normothermic *Ex-Vivo* Lung Perfusion. Ann Thorac Surg (2011) 92(2):478–84. doi: 10.1016/j.athoracsur.2011.04.027 21704971

[42] Chen Q Yu W Shi J Shen J Hu Y Gong J. The Effect of Extracorporeal Membrane Oxygenation Therapy on Systemic Oxidative Stress Injury in a Porcine Model. Artif Organs (2014) 38(5):426–31. doi: 10.1111/aor.12198 24117786

[43] Wang S Krawiec C Patel S Kunselman AR Song J Lei F. Laboratory Evaluation of Hemolysis and Systemic Inflammatory Response in Neonatal Nonpulsatile and Pulsatile Extracorporeal Life Support Systems. Artif Organs (2015) 39(9):774–81. doi: 10.1111/aor.12466 25940752

[44] Passmore MR Fung YL Simonova G Foley SR Dunster KR Diab SD. Inflammation and Lung Injury in an Ovine Model of Extracorporeal Membrane Oxygenation Support. Am J Physiol Lung Cell Mol Physiol (2016) 311(6):L1202–l12. doi: 10.1152/ajplung.00296.2016 27815258

[45] Thangappan K Cavarocchi NC Baram M Thoma B Hirose H. Systemic Inflammatory Response Syndrome (SIRS) After Extracorporeal Membrane Oxygenation (ECMO): Incidence, Risks and Survivals. Heart Lung J Crit Care (2016) 45(5):449–53. doi: 10.1016/j.hrtlng.2016.06.004 27425197

[46] Ciapetti M Mancinelli P Cecchi A Borrelli E Bocci V Peris A. Reduction of Non-Enzymatic Antioxidants in Plasma During ECMO-Treatment in ARDS by Influence A H1n1. J Crit Care (2018) 43:220–4. doi: 10.1016/j.jcrc.2017.08.005 28923478

[47] Masuodi S Blackwell J Stewart P Egan TM. Cytokine Levels in Steen Solution Perfusate Increase During *Ex-Vivo* Lung Perfusion (EVLP) of Lungs From Conventional Donors (Conv) and Uncontrolled Donation After Circulatory Determination of Death Donors (uDCDDs). J Heart Lung Transplant (2017) 36(4):S311–S2. doi: 10.1016/j.healun.2017.01.1508

[48] Lonati C Bassani GA Brambilla D Leonardi P Carlin A Faversani A. Influence of *Ex Vivo* Perfusion on the Biomolecular Profile of Rat Lungs. FASEB J (2018) 32(10):5532–49. doi: 10.1096/fj.201701255R 29718705

[49] Dikme R. Oxidative Stress and DNA Damage During Cardiopulmonary Bypass. Ann Clin Anal Med (2020) 11:1–5. doi: 10.4328/acam.5883

[50] Gao F Gao E Yue TL Ohlstein EH Lopez BL Christopher TA. Nitric Oxide Mediates the Antiapoptotic Effect of Insulin in Myocardial Ischemia-Reperfusion: The Roles of PI3-Kinase, Akt, and Endothelial Nitric Oxide Synthase Phosphorylation. Circulation (2002) 105(12):1497–502. doi: 10.1161/01.CIR.0000012529.00367.0F 11914261

[51] Holmes JHT Connolly NC Paull DL Hill ME Guyton SW Ziegler SF. Magnitude of the Inflammatory Response to Cardiopulmonary Bypass and Its Relation to Adverse Clinical Outcomes. Inflammation Res Off J Eur Histamine Res Soc [et al] (2002) 51(12):579–86. doi: 10.1007/PL00012432 12558191

[52] Kotani N Hashimoto H Sessler DI Muraoka M Wang JS O'Connor MF. Neutrophil Number and Interleukin-8 and Elastase Concentrations in Bronchoalveolar Lavage Fluid Correlate With Decreased Arterial Oxygenation After Cardiopulmonary Bypass. Anesth Analg (2000) 90(5):1046–51. doi: 10.1097/00000539-200005000-00009 10781451

[53] Fortenberry JD Bhardwaj V Niemer P Cornish JD Wright JA Bland L. Neutrophil and Cytokine Activation With Neonatal Extracorporeal Membrane Oxygenation. J Pediatr (1996) 128(5 Pt 1):670–8. doi: 10.1016/S0022-3476(96)80133-8 8627440

[54] Risnes I Wagner K Ueland T Mollnes T Aukrust P Svennevig J. Interleukin-6 May Predict Survival in Extracorporeal Membrane Oxygenation Treatment. Perfusion (2008) 23(3):173–8. doi: 10.1177/0267659108097882 19029268

[55] Noda K Tane S Haam SJ D’Cunha J Hayanga AJ Luketich JD. Targeting Circulating Leukocytes and Pyroptosis During *Ex Vivo* Lung Perfusion Improves Lung Preservation. Transplantation (2017) 101(12):2841–9. doi: 10.1097/TP.0000000000001798 28452921

[56] Aboelnazar NS Himmat S Hatami S White CW Burhani MS Dromparis P. Negative Pressure Ventilation Decreases Inflammation and Lung Edema During Normothermic Ex-Vivo Lung Perfusion. J Heart Lung Transplant Off Publ Int Soc Heart Transplant (2018) 37(4):520–30. doi: 10.1016/j.healun.2017.09.007 29103845

[57] Church JT Alghanem F Deatrick KB Trahanas JM Phillips JP Hee Song M. Normothermic *Ex Vivo* Heart Perfusion: Effects of Live Animal Blood and Plasma Cross Circulation. ASAIO J (American Soc Artif Internal Organs 1992) (2017) 63(6):766–73. doi: 10.1097/MAT.0000000000000583 PMC5630486 28394815

[58] McLeod JS Poling C Church JT Jung J Sarosi E Langley M. *Ex Vivo* Heart Perfusion for 72 Hours Using Plasma Cross Circulation. ASAIO J (American Soc Artif Internal Organs 1992) (2020) 66(7):753–9. doi: 10.1097/MAT.0000000000001061 31453833

[59] Hozain AE O’Neill JD Pinezich MR Tipograf Y Donocoff R Cunningham KM. Xenogeneic Cross-Circulation for Extracorporeal Recovery of Injured Human Lungs. Nat Med (2020) 26(7):1102–13. doi: 10.1038/s41591-020-0971-8 PMC9990469 32661401

[60] Seewald M Coles JA Jr. Sigg DC Iaizzo PA. Featured Article: Pharmacological Postconditioning With Delta Opioid Attenuates Myocardial Reperfusion Injury in Isolated Porcine Hearts. Exp Biol Med (Maywood NJ) (2017) 242(9):986–95. doi: 10.1177/1535370216684041 PMC5407584 28440739

[61] Sandha JK White CW Muller A Avery E Thliveris J Dixon IMC. Steroids Limit Myocardial Edema During *Ex Vivo* Perfusion of Hearts Donated After Circulatory Death. Ann Thorac Surg (2018) 105(6):1763–70. doi: 10.1016/j.athoracsur.2018.01.004 29382512

[62] Iyer A Gao L Doyle A Rao P Jayewardene D Wan B. Increasing the Tolerance of DCD Hearts to Warm Ischemia by Pharmacological Postconditioning. Am J Transplant Off J Am Soc Transplant Am Soc Transplant Surg (2014) 14(8):1744–52. doi: 10.1111/ajt.12782 25040306

[63] Qi X Hatami S Bozso S Khan M Himmat S Forgie K. Reduced Flow During *Ex-Situ* Heart Perfusion Provides Superior Function Preservation and Less Edema Formation. J Heart Lung Transplant (2021) 40(4):S14. doi: 10.1016/j.healun.2021.01.1770

[64] Zakkar M Guida G Suleiman MS Angelini GD. Cardiopulmonary Bypass and Oxidative Stress. Oxid Med Cell Longevity (2015) 2015:189863. doi: 10.1155/2015/189863 PMC4334937 25722792

[65] Doyle AJ Hunt BJ. Current Understanding of How Extracorporeal Membrane Oxygenators Activate Haemostasis and Other Blood Components. Front Med (2018) 5:352. doi: 10.3389/fmed.2018.00352 PMC6299009 30619862

[66] Datzmann T Trager K. Extracorporeal Membrane Oxygenation and Cytokine Adsorption. J Thorac Dis (2018) 10(Suppl 5):S653–s60. doi: 10.21037/jtd.2017.10.128 PMC5911550 29732183

[67] Raffaeli G Ghirardello S Passera S Mosca F Cavallaro G. Oxidative Stress and Neonatal Respiratory Extracorporeal Membrane Oxygenation. Front Physiol (2018) 9(1739). doi: 10.3389/fphys.2018.01739 PMC6288438 30564143

[68] Ranucci M Baryshnikova E. Inflammation and Coagulation Following Minimally Invasive Extracorporeal Circulation Technologies. J Thorac Dis (2019) 11(Suppl 10):S1480–s8. doi: 10.21037/jtd.2019.01.27 PMC6586586 31293797

[69] Graulich J Sonntag J Marcinkowski M Bauer K Kossel H Buhrer C. Complement Activation by *In Vivo* Neonatal and *In Vitro* Extracorporeal Membrane Oxygenation. Mediators Inflammation (2002) 11(2):69–73. doi: 10.1080/09629350220131908 PMC1781648 12061426

[70] Vallhonrat H Swinford RD Ingelfinger JR Williams WW Ryan DP Tolkoff-Rubin N. Rapid Activation of the Alternative Pathway of Complement by Extracorporeal Membrane Oxygenation. ASAIO J (American Soc Artif Internal Organs 1992) (1999) 45(1):113–4. doi: 10.1097/00002480-199901000-00025 9952020

[71] Gemmell CH Ramirez SM Yeo EL Sefton MV. Platelet Activation in Whole Blood by Artificial Surfaces: Identification of Platelet-Derived Microparticles and Activated Platelet Binding to Leukocytes as Material-Induced Activation Events. J Lab Clin Med (1995) 125(2):276–87. 7531214

[72] Gorbet MB Sefton MV. Biomaterial-Associated Thrombosis: Roles of Coagulation Factors, Complement, Platelets and Leukocytes. Biomaterials (2004) 25(26):5681–703. doi: 10.1016/j.biomaterials.2004.01.023 15147815

[73] Weerasinghe A Taylor KM. The Platelet in Cardiopulmonary Bypass. Ann Thorac Surg (1998) 66(6):2145–52. doi: 10.1016/S0003-4975(98)00749-8 9930521

[74] Rinder CS Gaal D Student LA Smith BR. Platelet-Leukocyte Activation and Modulation of Adhesion Receptors in Pediatric Patients With Congenital Heart Disease Undergoing Cardiopulmonary Bypass. J Thorac Cardiovasc Surg (1994) 107(1):280–8. doi: 10.1016/S0022-5223(94)70482-1 8283897

[75] Kraft F Schmidt C Van Aken H Zarbock A. Inflammatory Response and Extracorporeal Circulation. Best Pract Res Clin Anaesthesiol (2015) 29(2):113–23. doi: 10.1016/j.bpa.2015.03.001 26060024

[76] Passaroni AC Felicio ML Campos N Silva MAM Yoshida WB. Hemolysis and Inflammatory Response to Extracorporeal Circulation During On-Pump CABG: Comparison Between Roller and Centrifugal Pump Systems. Braz J Cardiovasc Surg (2018) 33(1):64–71. doi: 10.21470/1678-9741-2017-0125 29617504 PMC5873773

[77] Keyser A Hilker MK Diez C Philipp A Foltan M Schmid C. Prospective Randomized Clinical Study of Arterial Pumps Used for Routine on Pump Coronary Bypass Grafting. Artif Organs (2011) 35(5):534–42. doi: 10.1111/j.1525-1594.2010.01120.x 21269302

[78] Van Avondt K Nur E Zeerleder S. Mechanisms of Haemolysis-Induced Kidney Injury. Nat Rev Nephrol (2019) 15(11):671–92. doi: 10.1038/s41581-019-0181-0 31455889

[79] Ricci Z. Hemolysis During Pediatric Cardiac Surgery: An Old Issue With Renewed Concerns. J Lab Precis Med (2018) 3(2). doi: 10.21037/jlpm.2018.01.13

[80] Karsten E Breen E Herbert BR. Red Blood Cells Are Dynamic Reservoirs of Cytokines. Sci Rep (2018) 8(1):3101. doi: 10.1038/s41598-018-21387-w 29449599 PMC5814557

[81] Vermeulen Windsant IC Hanssen SJ Buurman WA Jacobs MJ. Cardiovascular Surgery and Organ Damage: Time to Reconsider the Role of Hemolysis. J Thorac Cardiovasc Surg (2011) 142(1):1–11. doi: 10.1016/j.jtcvs.2011.02.012 21570697

[82] Mariani AW Pego-Fernandes PM Abdalla LG Jatene FB. *Ex Vivo* Lung Reconditioning: A New Era for Lung Transplantation. J Bras Pneumol Publicacao Oficial Soc Bras Pneumol Tisilogia (2012) 38(6):776–85. doi: 10.1590/s1806-37132012000600015 23288125

[83] Wu ZJ Gartner M Litwak KN Griffith BP. Progress Toward an Ambulatory Pump-Lung. J Thorac Cardiovasc Surg (2005) 130(4):973–8. doi: 10.1016/j.jtcvs.2005.04.032 16214507

[84] Cypel M Yeung JC Hirayama S Rubacha M Fischer S Anraku M. Technique for Prolonged Normothermic *Ex Vivo* Lung Perfusion. J Heart Lung Transplant (2008) 27(12):1319–25. doi: 10.1016/j.healun.2008.09.003 19059112

[85] Kappler B Ledezma CA van Tuijl S Meijborg V Boukens BJ Ergin B. Investigating the Physiology of Normothermic *Ex Vivo* Heart Perfusion in an Isolated Slaughterhouse Porcine Model Used for Device Testing and Training. BMC Cardiovasc Disord (2019) 19(1):254. doi: 10.1186/s12872-019-1242-9 31711426 PMC6849278

[86] Rinder CS Rinder HM Smith MJ Fitch JC Tracey JB Chandler WL. Antithrombin Reduces Monocyte and Neutrophil CD11b Up Regulation in Addition to Blocking Platelet Activation During Extracorporeal Circulation. Transfusion (2006) 46(7):1130–7. doi: 10.1111/j.1537-2995.2006.00861.x 16836559

[87] Rinder CS Rinder HM Johnson K Smith M Lee DL Tracey J. Role of C3 Cleavage in Monocyte Activation During Extracorporeal Circulation. Circulation (1999) 100(5):553–8. doi: 10.1161/01.CIR.100.5.553 10430771

[88] Farneti PA Sbrana S Spiller D Cerillo AG Santarelli F Di Dario D. Reduction of Blood Coagulation and Monocyte-Platelet Interaction Following the Use of a Minimal Extracorporeal Circulation System (Synergy) in Coronary Artery Bypass Grafting (CABG). Perfusion (2008) 23(1):49–56. doi: 10.1177/0267659108091336 18788218

[89] Sbrana S Parri MS De Filippis R Gianetti J Clerico A. Monitoring of Monocyte Functional State After Extracorporeal Circulation: A Flow Cytometry Study. Cytometry Part B Clin Cytometry (2004) 58(1):17–24. doi: 10.1002/cyto.b.10061 14994371

[90] Patrignani P Santini G Panara MR Sciulli MG Greco A Rotondo MT. Induction of Prostaglandin Endoperoxide Synthase-2 in Human Monocytes Associated With Cyclo-Oxygenase-Dependent F2-Isoprostane Formation. Br J Pharmacol (1996) 118(5):1285–93. doi: 10.1111/j.1476-5381.1996.tb15535.x PMC1909586 8818355

[91] Mossanen JC Jansen TU Pracht J Liepelt A Buendgens L Stoppe C. Elevated Circulating CD14(++)CD16(+) Intermediate Monocytes are Independently Associated With Extracardiac Complications After Cardiac Surgery. Sci Rep (2020) 10(1):947. doi: 10.1038/s41598-020-57700-9 31969629 PMC6976615

[92] Philippidis P Athanasiou T Nadra I Ashrafian H Haskard DO Landis RC. Anti-Inflammatory Haemoglobin Scavenging Monocytes Are Induced Following Coronary Artery Bypass Surgery. Eur J Cardio-Thorac Surg Off J Eur Assoc Cardio-Thorac Surg (2010) 37(6):1360–6. doi: 10.1016/j.ejcts.2009.12.043 20153663

[93] Graulich J Walzog B Marcinkowski M Bauer K Kössel H Fuhrmann G. Leukocyte and Endothelial Activation in a Laboratory Model of Extracorporeal Membrane Oxygenation (ECMO). Pediatr Res (2000) 48(5):679–84. doi: 10.1203/00006450-200011000-00021 11044491

[94] Perttila J Salo M Pirttikangas CO Jalonen J Vainio O. Effects of Cardiopulmonary Bypass on Lymphocytes and Their Subset Counts With or Without Use of Autotransfusion Devices. J Cardiothorac Vasc Anesth (1994) 8(5):532–5. doi: 10.1016/1053-0770(94)90165-1 7803741

[95] Franke A Lante W Kurig E Zoller LG Weinhold C Markewitz A. Hyporesponsiveness of T Cell Subsets After Cardiac Surgery: A Product of Altered Cell Function or Merely a Result of Absolute Cell Count Changes in Peripheral Blood? Eur J Cardio-Thorac Surg Off J Eur Assoc Cardio-Thorac Surg (2006) 30(1):64–71. doi: 10.1016/j.ejcts.2006.03.029 16730447

[96] Vitkova V Panek M Janec P Sibikova M Vobruba V Haluzik M. Endothelial Microvesicles and Soluble Markers of Endothelial Injury in Critically Ill Newborns. Mediators of Inflammation (2018) 2018:1975056. doi: 10.1155/2018/1975056 30116143 PMC6079510

[97] Giacinto O Satriano U Nenna A Spadaccio C Lusini M Mastroianni C. Inflammatory Response and Endothelial Dysfunction Following Cardiopulmonary Bypass: Pathophysiology and Pharmacological Targets. Recent Pat Inflammation Allergy Drug Discovery (2019) 13(2):158–73. doi: 10.2174/1872213X13666190724112644 31339081

[98] Jacob-Ferreira AL Schulz R. Activation of Intracellular Matrix Metalloproteinase-2 by Reactive Oxygen-Nitrogen Species: Consequences and Therapeutic Strategies in the Heart. Arch Biochem Biophys (2013) 540(1-2):82–93. doi: 10.1016/j.abb.2013.09.019 24103691

[99] Radi R. Protein Tyrosine Nitration: Biochemical Mechanisms and Structural Basis of Functional Effects. Acc Chem Res (2013) 46(2):550–9. doi: 10.1021/ar300234c PMC3577981 23157446

[100] Hashimoto K Cypel M Kim H Machuca TN Nakajima D. Soluble Adhesion Molecules During *Ex Vivo* Lung Perfusion Are Associated With Posttransplant Primary Graft Dysfunction. Am J Transplant (2017) 17: (5):1396–404. doi: 10.1111/ajt.14160 27977885

[101] Sladden TM Yerkovich S. Endothelial Glycocalyx Shedding Occurs During *Ex Vivo* Lung Perfusion: A Pilot Study. J Transplant (2019) 2019:6748242. doi: 10.1155/2019/6748242 31534794 PMC6732651

[102] Andreasson AS Karamanou DM Gillespie CS Ozalp F Butt T Hill P. Profiling Inflammation and Tissue Injury Markers in Perfusate and Bronchoalveolar Lavage Fluid During Human *Ex Vivo* Lung Perfusion. Eur J Cardio-Thorac Surg Off J Eur Assoc Cardio-Thorac Surg (2017) 51(3):577–86. doi: 10.1093/ejcts/ezw358 PMC5400024 28082471

[103] Bown MJ Nicholson ML Bell PR Sayers RD. Cytokines and Inflammatory Pathways in the Pathogenesis of Multiple Organ Failure Following Abdominal Aortic Aneurysm Repair. Eur J Vasc Endovasc Surg Off J Eur Soc Vasc Surg (2001) 22(6):485–95. doi: 10.1053/ejvs.2001.1522 11735196

[104] Zhang JM An J. Cytokines, Inflammation, and Pain. Int Anesthesiol Clinics (2007) 45(2):27–37. doi: 10.1097/AIA.0b013e318034194e PMC2785020 17426506

[105] Laffey JG Boylan JF Cheng DC. The Systemic Inflammatory Response to Cardiac Surgery: Implications for the Anesthesiologist. Anesthesiology (2002) 97(1):215–52. doi: 10.1097/00000542-200207000-00030 12131125

[106] Baumann A Buchwald D Annecke T Hellmich M Zahn PK Hohn A. RECCAS - REmoval of Cytokines During CArdiac Surgery: Study Protocol for a Randomised Controlled Trial. Trials (2016) 17(1):137. doi: 10.1186/s13063-016-1265-9 26971164 PMC4789286

[107] Bernardi MH Rinoesl H Dragosits K Ristl R Hoffelner F Opfermann P. Effect of Hemoadsorption During Cardiopulmonary Bypass Surgery – A Blinded, Randomized, Controlled Pilot Study Using a Novel Adsorbent. Crit Care (2016) 20(1):96. doi: 10.1186/s13054-016-1270-0 27059056 PMC4826492

[108] Poli EC Alberio L Bauer-Doerries A Marcucci C Roumy A Kirsch M. Cytokine Clearance With CytoSorb® During Cardiac Surgery: A Pilot Randomized Controlled Trial. Crit Care (2019) 23(1):108. doi: 10.1186/s13054-019-2399-4 30944029 PMC6448322

[109] Taleska Stupica G Sostaric M Bozhinovska M Rupert L Bosnic Z Jerin A. Extracorporeal Hemadsorption Versus Glucocorticoids During Cardiopulmonary Bypass: A Prospective, Randomized, Controlled Trial. Cardiovascular Therapeutics (2020) 2020:7834173. doi: 10.1053/j.jvca.2020.09.035 32292492 PMC7149340

[110] Nemeth E Kovacs E Racz K Soltesz A Szigeti S Kiss N. Impact of Intraoperative Cytokine Adsorption on Outcome of Patients Undergoing Orthotopic Heart Transplantation-an Observational Study. Clin Transplant (2018) 32(4):e13211. doi: 10.1111/ctr.13211 29377282

[111] Bruenger F Kizner L Weile J Morshuis M Gummert JF. First Successful Combination of ECMO With Cytokine Removal Therapy in Cardiogenic Septic Shock: A Case Report. Int J Artif Organs (2015) 38(2):113–6. doi: 10.5301/ijao.5000382 25656010

[112] Lother A Benk C Staudacher DL Supady A Bode C Wengenmayer T. Cytokine Adsorption in Critically Ill Patients Requiring ECMO Support. Front Cardiovasc Med (2019) 6:71. doi: 10.3389/fcvm.2019.00071 31275944 PMC6593298

[113] Magoon R Loona M Kohli JK Kashav R. Cytokine Adsorption in Cardiac Surgery: Where Do We Stand? Braz J Cardiovasc Surg (2020) 35(3):Xv–xvi. doi: 10.21470/1678-9741-2019-0480 32549093 PMC7299579

[114] Spoelstra-de Man AM Smit B Oudemans-van Straaten HM Smulders YM. Cardiovascular Effects of Hyperoxia During and After Cardiac Surgery. Anaesthesia (2015) 70(11):1307–19. doi: 10.1111/anae.13218 26348878

[115] Caputo M Mokhtari A Miceli A Ghorbel MT Angelini GD Parry AJ. Controlled Reoxygenation During Cardiopulmonary Bypass Decreases Markers of Organ Damage, Inflammation, and Oxidative Stress in Single-Ventricle Patients Undergoing Pediatric Heart Surgery. J Thorac Cardiovasc Surg (2014) 148(3):792–801.e8; discussion 0-1. doi: 10.1016/j.jtcvs.2014.06.001 25052821

[116] Caputo M Mokhtari A Rogers CA Panayiotou N Chen Q Ghorbel MT. The Effects of Normoxic Versus Hyperoxic Cardiopulmonary Bypass on Oxidative Stress and Inflammatory Response in Cyanotic Pediatric Patients Undergoing Open Cardiac Surgery: A Randomized Controlled Trial. J Thorac Cardiovasc Surg (2009) 138(1):206–14. doi: 10.1016/j.jtcvs.2008.12.028 PMC2840834 19577081

[117] Hayes RA Shekar K Fraser JF. Is Hyperoxaemia Helping or Hurting Patients During Extracorporeal Membrane Oxygenation? Rev Complex Probl Perfusion (2013) 28(3):184–93. doi: 10.1177/0267659112473172 23322670

[118] Trittenwein G Rotta AT Gunnarsson B Steinhorn DM. Lipid Peroxidation During Initiation of Extracorporeal Membrane Oxygenation After Hypoxia in Endotoxemic Rabbits. Perfusion (1999) 14(1):49–57. doi: 10.1177/026765919901400108 10074647

[119] Fujii Y Tatsumi E Nakamura F Oite T. PaO 2 Greater Than 300 mmHg Promotes an Inflammatory Response During Extracorporeal Circulation in a Rat Extracorporeal Membrane Oxygenation Model. J Thorac Dis (2020) 12(3):749–57. doi: 10.21037/jtd.2019.12.113 PMC7139026 32274141

[120] Sznycer-Taub NR Lowery R Yu S Owens ST Hirsch-Romano JC Owens GE. Hyperoxia Is Associated With Poor Outcomes in Pediatric Cardiac Patients Supported on Venoarterial Extracorporeal Membrane Oxygenation. Pediatr Crit Care Med J Soc Crit Care Med World Fed Pediatr Intensive Crit Care Soc (2016) 17(4):350–8. doi: 10.1097/PCC.0000000000000655 27043897

[121] Cashen K Reeder R Dalton HJ Berg RA Shanley TP Newth CJL. Hyperoxia and Hypocapnia During Pediatric Extracorporeal Membrane Oxygenation: Associations With Complications, Mortality, and Functional Status Among Survivors. Pediatr Crit Care Med J Soc Crit Care Med World Fed Pediatr Intensive Crit Care Soc (2018) 19(3):245–53. doi: 10.1097/PCC.0000000000001439 PMC5834382 29319634

[122] Kuzmiak-Glancy S Jaimes R 3rd Wengrowski AM Kay MW. Oxygen Demand of Perfused Heart Preparations: How Electromechanical Function and Inadequate Oxygenation Affect Physiology and Optical Measurements. Exp Physiol (2015) 100(6):603–16. doi: 10.1113/EP085042 PMC4506772 25865254

[123] White CW Hasanally D Mundt P Li Y Klein J Xiang B. A Wholeblood–Based Perfusate Provides Superior Preservation of Myocardial Function During *Ex Vivo* Heart Perfusion. J Heart Lung Transplant Off Publ Int Soc Heart Transplant (2014) 34:S1053–2498(14):01356-4. doi: 10.1016/j.healun.2014.09.021 25447577

[124] White CW Ali A Hasanally D Xiang B Li Y Mundt P. A Cardioprotective Preservation Strategy Employing *Ex Vivo* Heart Perfusion Facilitates Successful Transplant of Donor Hearts After Cardiocirculatory Death. J Heart Lung Transplant Off Publ Int Soc Heart Transplant (2013) 32(7):734–43. doi: 10.1016/j.healun.2013.04.016 23796155

[125] Noda K Tane S Haam SJ Hayanga AJ D’Cunha J Luketich JD. Optimal *Ex Vivo* Lung Perfusion Techniques With Oxygenated Perfusate. J Heart Lung Transplant (2017) 36(4):466–74. doi: 10.1016/j.healun.2016.10.014 27914896

[126] Sanada S Komuro I Kitakaze M. Pathophysiology of Myocardial Reperfusion Injury: Preconditioning, Postconditioning, and Translational Aspects of Protective Measures. Am J Physiol Heart Circulatory Physiol (2011) 301(5):H1723–41. doi: 10.1152/ajpheart.00553.2011 21856909

[127] Soares ROS Losada DM Jordani MC Evora P Castro ESO. Ischemia/reperfusion Injury Revisited: An Overview of the Latest Pharmacological Strategies. Int J Mol Sci (2019) 20(20):5034. doi: 10.3390/ijms20205034 PMC6834141 31614478

[128] Salameh A Dhein S. Strategies for Pharmacological Organoprotection During Extracorporeal Circulation Targeting Ischemia-Reperfusion Injury. Front Pharmacol (2015) 6:296. doi: 10.3389/fphar.2015.00296 26733868 PMC4686733

[129] Clermont G Vergely C Jazayeri S Lahet JJ Goudeau JJ Lecour S. Systemic Free Radical Activation Is a Major Event Involved in Myocardial Oxidative Stress Related to Cardiopulmonary Bypass. Anesthesiology (2002) 96(1):80–7. doi: 10.1097/00000542-200201000-00019 11753006

[130] White CW Messer SJ Large SR Conway J Kim DH Kutsogiannis DJ. Transplantation of Hearts Donated After Circulatory Death. Front Cardiovasc Med (2018) 5:8. doi: 10.3389/fcvm.2018.00008 29487855 PMC5816942

[131] Charles EJ Hunter Mehaffey J Huerter ME Sharma AK Stoler MH Roeser ME. *Ex Vivo* Assessment of Porcine Donation After Circulatory Death Lungs That Undergo Increasing Warm Ischemia Times. Transplant Direct (2018) 4(12):e405. doi: 10.1097/TXD.0000000000000845 30584586 PMC6283086

[132] Estensen K Shekar K Robins E McDonald C Barnett AG Fraser JF. Macro- and Micronutrient Disposition in an *Ex Vivo* Model of Extracorporeal Membrane Oxygenation. Intensive Care Med Exp (2014) 2(1):29. doi: 10.1186/s40635-014-0029-7 26266926 PMC4512975

[133] McDonald CI Fung YL Fraser JF. Antioxidant Trace Element Reduction in an *In Vitro* Cardiopulmonary Bypass Circuit. ASAIO J (American Soc Artif Internal Organs 1992) (2012) 58(3):217–22. doi: 10.1097/MAT.0b013e31824cc856 22460776

[134] Yan YQ Liu XC Jing WB Wang Z Bai XY Yang Q. Alteration of Plasma Trace Elements in Patients Undergoing Open Heart Surgery. Biol Trace Elem Res (2013) 151(3):344–9. doi: 10.1007/s12011-012-9577-4 23264034

[135] Buchko MT Stewart CJ. Total Parenteral Nutrition in *Ex Vivo* Lung Perfusion: Addressing Metabolism Improves Both Inflammation and Oxygenation. Am J Transplant (2019) 19(12):3390–7. doi: 10.1111/ajt.15572 31420938

[136] Qi X Hatami S White C Himmat S Aboelnazar N Ondrus M. Inflammation and Innate Immune Responses Activation During *Ex Vivo* Heart Perfusion. J Heart Lung Transplant Off Publ Int Soc Heart Transplant (2018) 37(4):s220. doi: 10.1016/j.healun.2018.01.543

[137] Andreasson ASI Karamanou DM Gillespie CS Özalp F Butt T Hill P. Profiling Inflammation and Tissue Injury Markers in Perfusate and Bronchoalveolar Lavage Fluid During Human *Ex Vivo* Lung Perfusion. Eur J Cardio-Thorac Surg (2016) 51(3):577–86. doi: 10.1016/j.healun.2016.01.241 PMC5400024 28082471

[138] Sage AT Richard-Greenblatt M Zhong K Bai XH Snow MB Babits M. Prediction of Donor Related Lung Injury in Clinical Lung Transplantation Using a Validated *Ex Vivo* Lung Perfusion Inflammation Score. J Heart Lung Transplant (2021) 40(7):687–95. doi: 10.1016/j.healun.2021.03.002 33781664

[139] Hatami S Qi X White C Buchko M Bozso S Himmat S. Oxidative Stress and Related Responses are Induced During *Ex-Situ* Heart Perfusion and may Contribute to the Functional Decline of the *Ex-Situ*-Perfused Heart. Can J Cardiol (2019) 35(10):S106. doi: 10.1016/j.cjca.2019.07.499

[140] Hatami S Qi X Bozso S Khan M Tkachuk B Himmat S. INFLAMMATION, OXIDATIVE STRESS AND FUNCTIONAL DECLINE OF THE HEART DURING *EX SITU* HEART PERFUSION: ARE LEUKOCYTES THE ULTIMATE VILLAINS? Can J Cardiol (2020) 36(10):S69. doi: 10.1016/j.cjca.2020.07.140

[141] Lu J Xu L Zeng Z Xue C Li J Chen X. Normothermic *Ex Vivo* Heart Perfusion Combined With Melatonin Enhances Myocardial Protection in Rat Donation After Circulatory Death Hearts *via* Inhibiting NLRP3 Inflammasome-Mediated Pyroptosis. Front Cell Dev Biol (2021) 9(2419). doi: 10.3389/fcell.2021.733183 PMC8438322 34532321

[142] Mahmood S Bilal H Zaman M Tang A. Is a Fully Heparin-Bonded Cardiopulmonary Bypass Circuit Superior to a Standard Cardiopulmonary Bypass Circuit? Interact Cardiovasc Thorac Surg (2012) 14(4):406–14. doi: 10.1093/icvts/ivr124 PMC3309813 22228288

[143] Mollnes TE Videm V Gotze O Harboe M Oppermann M. Formation of C5a During Cardiopulmonary Bypass: Inhibition by Precoating With Heparin. Ann Thorac Surg (1991) 52(1):92–7. doi: 10.1016/0003-4975(91)91426-V 2069469

[144] Ontaneda A Annich GM. Novel Surfaces in Extracorporeal Membrane Oxygenation Circuits. Front Med (2018) 5:321. doi: 10.3389/fmed.2018.00321 PMC6256321 30525038

[145] de Vroege R Huybregts R van Oeveren W van Klarenbosch J Linley G Mutlu J. The Impact of Heparin-Coated Circuits on Hemodynamics During and After Cardiopulmonary Bypass. Artif Organs (2005) 29(6):490–7. doi: 10.1111/j.1525-1594.2005.29083.x 15926987

[146] Fosse E Moen O Johnson E Semb G Brockmeier V Mollnes TE. Reduced Complement and Granulocyte Activation With Heparin-Coated Cardiopulmonary Bypass. Ann Thorac Surg (1994) 58(2):472–7. doi: 10.1016/0003-4975(94)92231-4 8067851

[147] Ranucci M Balduini A Ditta A Boncilli A Brozzi S. A Systematic Review of Biocompatible Cardiopulmonary Bypass Circuits and Clinical Outcome. Ann Thorac Surg (2009) 87(4):1311–9. doi: 10.1016/j.athoracsur.2008.09.076 19324190

[148] Sohn N Marcoux J Mycyk T Krahn J Meng Q. The Impact of Different Biocompatible Coated Cardiopulmonary Bypass Circuits on Inflammatory Response and Oxidative Stress. Perfusion (2009) 24(4):231–7. doi: 10.1177/0267659109351218 19858237

[149] Fransson F Kyrk T Skagerlind M Stegmayr B. Rinsing the Extra Corporeal Circuit With a Heparin and Albumin Solution Reduces the Need for Systemic Anticoagulant in Hemodialysis. Int J Artif Organs (2013) 36(10):725–9. doi: 10.5301/ijao.5000253 24254840

[150] Reynolds MM Annich GM. The Artificial Endothelium. Organogenesis (2011) 7(1):42–9. doi: 10.4161/org.7.1.14029 PMC3082033 21289481

[151] Wo Y Brisbois EJ Bartlett RH Meyerhoff ME. Recent Advances in Thromboresistant and Antimicrobial Polymers for Biomedical Applications: Just Say Yes to Nitric Oxide (NO). Biomater Sci (2016) 4(8):1161–83. doi: 10.1039/C6BM00271D PMC4955746 27226170

[152] De Somer F François K van Oeveren W Poelaert J De Wolf D Ebels T. Phosphorylcholine Coating of Extracorporeal Circuits Provides Natural Protection Against Blood Activation by the Material Surface. Eur J Cardio-Thorac Surg Off J Eur Assoc Cardio-Thorac Surg (2000) 18(5):602–6. doi: 10.1016/S1010-7940(00)00508-X 11053824

[153] Saito N Motoyama S Sawamoto J. Effects of New Polymer-Coated Extracorporeal Circuits on Biocompatibility During Cardiopulmonary Bypass. Artif Organs (2000) 24(7):547–54. doi: 10.1046/j.1525-1594.2000.06520.x 10916066

[154] Suzuki Y Daitoku K Minakawa M Fukui K Fukuda I. Poly-2-Methoxyethylacrylate-Coated Bypass Circuits Reduce Activation of Coagulation System and Inflammatory Response in Congenital Cardiac Surgery. J Artif Organs Off J Jpn Soc Artif Organs (2008) 11(3):111–6. doi: 10.1007/s10047-008-0415-6 18836870

[155] Vohra HA Whistance R Modi A Ohri SK. The Inflammatory Response to Miniaturised Extracorporeal Circulation: A Review of the Literature. Mediators Inflammation (2009) 2009:707042. doi: 10.1155/2009/707042 PMC2809242 20101278

[156] Immer FF Ackermann A Gygax E Stalder M Englberger L Eckstein FS. Minimal Extracorporeal Circulation Is a Promising Technique for Coronary Artery Bypass Grafting. Ann Thorac Surg (2007) 84(5):1515–20; discussion 21. doi: 10.1016/j.athoracsur.2007.05.069 17954055

[157] Williams DC Turi JL Hornik CP Bonadonna DK Williford WL Walczak RJ. Circuit Oxygenator Contributes to Extracorporeal Membrane Oxygenation-Induced Hemolysis. ASAIO J (American Soc Artif Internal Organs 1992) (2015) 61(2):190–5. doi: 10.1097/MAT.0000000000000173 PMC4537148 25419829

[158] Nelson K Bobba C Ghadiali S Hayes D Jr. Black SM Whitson BA. Animal Models of *Ex Vivo* Lung Perfusion as a Platform for Transplantation Research. World J Exp Med (2014) 4(2):7–15. doi: 10.5493/wjem.v4.i2.7 24977117 PMC4073219

[159] Makdisi G Makdisi T Jarmi T Caldeira CC. *Ex Vivo* Lung Perfusion Review of a Revolutionary Technology. Ann Trans Med (2017) 5(17):343. doi: 10.21037/atm.2017.07.17 PMC5599284 28936437

[160] Day JR Taylor KM. The Systemic Inflammatory Response Syndrome and Cardiopulmonary Bypass. Int J Surg (London England) (2005) 3(2):129–40. doi: 10.1016/j.ijsu.2005.04.002 17462274

[161] de Amorim CG Malbouisson LM da Silva FC Jr. Fiorelli AI Murakami CK Carmona MJ. Leukocyte Depletion During CPB: Effects on Inflammation and Lung Function. Inflammation (2014) 37(1):196–204. doi: 10.1007/s10753-013-9730-z 24092406 PMC3929029

[162] Karaiskos TE Palatianos GM Triantafillou CD Kantidakis GH Astras GM Papadakis EG. Clinical Effectiveness of Leukocyte Filtration During Cardiopulmonary Bypass in Patients With Chronic Obstructive Pulmonary Disease. Ann Thorac Surg (2004) 78(4):1339–44. doi: 10.1016/j.athoracsur.2004.04.040 15464496

[163] Boodram S Evans E. Use of Leukocyte-Depleting Filters During Cardiac Surgery With Cardiopulmonary Bypass: A Review. J Extra-corporeal Technol (2008) 40(1):27–42. PMC4680653 18389663

[164] Dvorak L Pirk J Cerny S Kovar J. The Role of Leukocyte Depleting Filters in Heart Transplantation: Early Outcomes in Prospective, Randomized Clinical Trial. Eur J Cardio-Thorac Surg (2006) 30(4):621–7. doi: 10.1016/j.ejcts.2006.07.022 16949830

[165] Jackson HT Oyetunji TA Thomas A Oyetunji AO Hamrick M Nadler EP. The Impact of Leukoreduced Red Blood Cell Transfusion on Mortality of Neonates Undergoing Extracorporeal Membrane Oxygenation. J Surg Res (2014) 192(1):6–11. doi: 10.1016/j.jss.2014.06.013 25033708

[166] Canter MO Daniels J Bridges BC. Adjunctive Therapies During Extracorporeal Membrane Oxygenation to Enhance Multiple Organ Support in Critically Ill Children. Front Pediatr (2018) 2018(6):78. doi: 10.3389/fped.2018.00078 PMC5893897 29670870

[167] Tang J Tao K Zhou J Zhang C Gong L Luo N. Long-Term Leukocyte Filtration Should be Avoided During Extracorporeal Circulation. Mediators Inflammation (2013) 2013:612848. doi: 10.1155/2013/612848 PMC3888746 24453424

[168] Luc JGY Aboelnazar NS Himmat S Hatami S Haromy A Matsumura N. A Leukocyte Filter Does Not Provide Further Benefit During *Ex Vivo* Lung Perfusion. ASAIO J (American Soc Artif Internal Organs 1992) (2017) 63(5):672–8. doi: 10.1097/MAT.0000000000000550 28234641

[169] Messer S Ardehali A Tsui S. Normothermic Donor Heart Perfusion: Current Clinical Experience and the Future. Transplant Int Off J Eur Soc Organ Transplant (2015) 28(6):634–42. doi: 10.1111/tri.12361 24853906

[170] White CW Ambrose E Muller A Li Y Le H Hiebert B. Assessment of Donor Heart Viability During *Ex Vivo* Heart Perfusion. Can J Physiol Pharmacol (2015) 93(10):893–901. doi: 10.1139/cjpp-2014-0474 26317524

[171] Anic D Gasparovic H Ivancan V Batinic D. Effects of Corticosteroids on Inflammatory Response Following Cardiopulmonary Bypass. Croatian Med J (2004) 45(2):158–61. 15103751

[172] Celik JB Gormus N Okesli S Gormus ZI Solak H. Methylprednisolone Prevents Inflammatory Reaction Occurring During Cardiopulmonary Bypass: Effects on TNF-Alpha, IL-6, IL-8, IL-10. Perfusion (2004) 19(3):185–91. doi: 10.1191/0267659104pf733oa 15298427

[173] Giomarelli P Scolletta S Borrelli E Biagioli B. Myocardial and Lung Injury After Cardiopulmonary Bypass: Role of Interleukin (IL)-10. Ann Thorac Surg (2003) 76(1):117–23. doi: 10.1016/S0003-4975(03)00194-2 12842524

[174] Demir T Ergenoglu MU Demir HB Tanrikulu N Sahin M Gok E. Pretreatment With Methylprednisolone Improves Myocardial Protection During on-Pump Coronary Artery Bypass Surgery. Heart Surg Forum (2015) 18(4):E171–7. doi: 10.1532/hsf.1367 26334856

[175] Liakopoulos OJ Schmitto JD Kazmaier S Brauer A Quintel M Schoendube FA. Cardiopulmonary and Systemic Effects of Methylprednisolone in Patients Undergoing Cardiac Surgery. Ann Thorac Surg (2007) 84(1):110–8; discussion 8-9. doi: 10.1016/j.athoracsur.2007.01.003 17588396

[176] Fillinger MP Rassias AJ Guyre PM Sanders JH Beach M Pahl J. Glucocorticoid Effects on the Inflammatory and Clinical Responses to Cardiac Surgery. J Cardiothorac Vasc Anesth (2002) 16(2):163–9. doi: 10.1053/jcan.2002.31057 11957164

[177] Halonen J Halonen P Jarvinen O Taskinen P Auvinen T Tarkka M. Corticosteroids for the Prevention of Atrial Fibrillation After Cardiac Surgery: A Randomized Controlled Trial. Jama (2007) 297(14):1562–7. doi: 10.1001/jama.297.14.1562 17426275

[178] Kilger E Weis F Briegel J Frey L Goetz AE Reuter D. Stress Doses of Hydrocortisone Reduce Severe Systemic Inflammatory Response Syndrome and Improve Early Outcome in a Risk Group of Patients After Cardiac Surgery. Crit Care Med (2003) 31(4):1068–74. doi: 10.1097/01.CCM.0000059646.89546.98 12682474

[179] Bourbon A Vionnet M Leprince P Vaissier E Copeland J McDonagh P. The Effect of Methylprednisolone Treatment on the Cardiopulmonary Bypass-Induced Systemic Inflammatory Response. Eur J Cardio-Thorac Surg Off J Eur Assoc Cardio-Thorac Surg (2004) 26(5):932–8. doi: 10.1016/j.ejcts.2004.07.044 15519185

[180] Toft P Christiansen K Tonnesen E Nielsen CH Lillevang S. Effect of Methylprednisolone on the Oxidative Burst Activity, Adhesion Molecules and Clinical Outcome Following Open Heart Surgery. Scand Cardiovasc J SCJ (1997) 31(5):283–8. doi: 10.3109/14017439709069549 9406295

[181] Ho KM Tan JA. Benefits and Risks of Corticosteroid Prophylaxis in Adult Cardiac Surgery: A Dose-Response Meta-Analysis. Circulation (2009) 119(14):1853–66. doi: 10.1161/CIRCULATIONAHA.108.848218 19332460

[182] Whitlock RP Chan S Devereaux PJ Sun J Rubens FD Thorlund K. Clinical Benefit of Steroid Use in Patients Undergoing Cardiopulmonary Bypass: A Meta-Analysis of Randomized Trials. Eur Heart J (2008) 29(21):2592–600. doi: 10.1093/eurheartj/ehn333 18664462

[183] Robertson-Malt S Afrane B El Barbary M. Prophylactic Steroids for Pediatric Open Heart Surgery. The Cochrane Database of Systematic Reviews. Cochrane Database Systematic Reviews (2007) 2007(4):Cd005550. doi: 10.1002/14651858.CD005550.pub2 17943866

[184] Warren OJ Watret AL de Wit KL Alexiou C Vincent C Darzi AW. The Inflammatory Response to Cardiopulmonary Bypass: Part 2–Anti-Inflammatory Therapeutic Strategies. J Cardiothorac Vasc Anesth (2009) 23(3):384–93. doi: 10.1053/j.jvca.2008.09.007 19054695

[185] Meduri GU Bridges L Shih MC Marik PE Siemieniuk RAC Kocak M. Prolonged Glucocorticoid Treatment Is Associated With Improved ARDS Outcomes: Analysis of Individual Patients' Data From Four Randomized Trials and Trial-Level Meta-Analysis of the Updated Literature. Intensive Care Med (2016) 42(5):829–40. doi: 10.1007/s00134-015-4095-4 26508525

[186] Pappalardo F Montisci A. Adjunctive Therapies During Veno-Venous Extracorporeal Membrane Oxygenation. J Thorac Dis (2018) 10(Suppl 5):S683–s91. doi: 10.21037/jtd.2017.10.08 PMC5911547 29732187

[187] Ardehali A Esmailian F Deng M Soltesz E Hsich E Naka Y. Ex-Vivo Perfusion of Donor Hearts for Human Heart Transplantation (PROCEED II): A Prospective, Open-Label, Multicentre, Randomised Non-Inferiority Trial. Lancet (London England) (2015) 385(9987):2577–84. doi: 10.1016/S0140-6736(15)60261-6 25888086

[188] Van Raemdonck D Neyrinck A Cypel M Keshavjee S. *Ex-Vivo* Lung Perfusion. Transplant Int Off J Eur Soc Organ Transplant (2015) 28(6):643–56. doi: 10.1111/tri.12317 24629039

[189] van Zanden JE Leuvenink HGD Verschuuren EAM Veldhuis ZJ Ottens PJ Erasmus ME. *Ex Vivo* Perfusion With Methylprednisolone Attenuates Brain Death-Induced Lung Injury in Rats. Transplant Direct (2021) 7(4):e682. doi: 10.1097/TXD.0000000000001141 33748411 PMC7969243

[190] Martens A Boada M Vanaudenaerde BM Verleden SE Vos R Verleden GM. Steroids can Reduce Warm Ischemic Reperfusion Injury in a Porcine Donation After Circulatory Death Model With *Ex Vivo* Lung Perfusion Evaluation. Transplant Int Off J Eur Soc Organ Transplant (2016) 29(11):1237–46. doi: 10.1111/tri.12823 27514498

[191] Weir WB Hayes M Langley M Langley M Schneider B Drake D Rojas-Peña A. Steroid Use in Ex-Vivo Normothermic Heart Perfusion. San Francisco, CA: American Society for Artificial Internal Organs (2019). US2019.

[192] Mahalwar R Khanna D. Pleiotropic Antioxidant Potential of Rosuvastatin in Preventing Cardiovascular Disorders. Eur J Pharmacol (2013) 711(1-3):57–62. doi: 10.1016/j.ejphar.2013.04.025 23648561

[193] Morgan C Zappitelli M Gill P. Statin Prophylaxis and Inflammatory Mediators Following Cardiopulmonary Bypass: A Systematic Review. Crit Care (London England) (2009) 13(5):R165. doi: 10.1186/cc8135 PMC2784396 19840397

[194] Chee YR Watson RW McCarthy J Chughtai JZ Nolke L Healy DG. High Dose Statin Prophylaxis in Cardiopulmonary Bypass Related Surgery: Clinical Utility. J Cardiothorac Surg (2017) 12(1):20. doi: 10.1186/s13019-017-0582-8 28359339 PMC5374690

[195] An J Shi F Liu S Ma J Ma Q. Preoperative Statins as Modifiers of Cardiac and Inflammatory Outcomes Following Coronary Artery Bypass Graft Surgery: A Meta-Analysis. Interact Cardiovasc Thorac Surg (2017) 25(6):958–65. doi: 10.1093/icvts/ivx172 29049804

[196] Putzu A Capelli B Belletti A Cassina T Ferrari E Gallo M. Perioperative Statin Therapy in Cardiac Surgery: A Meta-Analysis of Randomized Controlled Trials. Crit Care (2016) 20(1):395. doi: 10.1186/s13054-016-1560-6 27919293 PMC5139027

[197] Putzu A de Carvalho e Silva CMPD de Almeida JP Belletti A Cassina T Landoni G. Perioperative Statin Therapy in Cardiac and Non-Cardiac Surgery: A Systematic Review and Meta-Analysis of Randomized Controlled Trials. Ann Intensive Care (2018) 8(1):95. doi: 10.1186/s13613-018-0441-3 30264290 PMC6160380

[198] Velez DE Mestre-Cordero VE Hermann R Perego J Harriet S Fernandez-Pazos MLM. Rosuvastatin Protects Isolated Hearts Against Ischemia-Reperfusion Injury: Role of Akt-GSK-3beta, Metabolic Environment, and Mitochondrial Permeability Transition Pore. J Physiol Biochem (2020) 76(1):85–98. doi: 10.1007/s13105-019-00718-z 31916218

[199] Liu CW Yang F Cheng SZ Liu Y Wan LH Cong HL. Rosuvastatin Postconditioning Protects Isolated Hearts Against Ischemia-Reperfusion Injury: The Role of Radical Oxygen Species, PI3K-Akt-GSK-3beta Pathway, and Mitochondrial Permeability Transition Pore. Cardiovasc Ther (2017) 35(1):3–9. doi: 10.1111/1755-5922.12225 27580017

[200] Hamamoto M Suga M Takahashi Y Sato Y Inamori S Yagihara T. Suppressive Effect of Phosphodiesterase Type 4 Inhibition on Systemic Inflammatory Responses After Cardiopulmonary Bypass. J Artif Organs Off J Jpn Soc Artif Organs (2006) 9(3):144–8. doi: 10.1007/s10047-006-0335-2 16998698

[201] Ustunsoy H Sivrikoz MC Tarakcioglu M Bakir K Guldur E Celkan MA. The Effects of Pentoxifylline on the Myocardial Inflammation and Ischemia-Reperfusion Injury During Cardiopulmonary Bypass. J Cardiac Surg (2006) 21(1):57–61. doi: 10.1111/j.1540-8191.2006.00169.x 16426349

[202] Yamaura K Akiyoshi K Irita K Taniyama T Takahashi S. Effects of Olprinone, a New Phosphodiesterase Inhibitor, on Gastric Intramucosal Acidosis and Systemic Inflammatory Responses Following Hypothermic Cardiopulmonary Bypass. Acta Anaesthesiol Scand (2001) 45(4):427–34. doi: 10.1034/j.1399-6576.2001.045004427.x 11300380

[203] Dabbagh A Rajaei S Bahadori Monfared A Keramatinia AA Omidi K. Cardiopulmonary Bypass, Inflammation and How to Defy It: Focus on Pharmacological Interventions. Iranian J Pharm Res IJPR (2012) 11(3):705–14. PMC3813123 24250497

[204] Wollborn J Siemering S Steiger C Buerkle H Goebel U Schick MA. Phosphodiesterase-4 Inhibition Reduces ECLS-Induced Vascular Permeability and Improves Microcirculation in a Rodent Model of Extracorporeal Resuscitation. Am J Physiol Heart Circulatory Physiol (2019) 316(3):H751–h61. doi: 10.1152/ajpheart.00673.2018 30681364

[205] Reignier J Mazmanian M Detruit H Chapelier A Weiss M Libert JM. Reduction of Ischemia-Reperfusion Injury by Pentoxifylline in the Isolated Rat Lung. Paris-Sud University Lung Transplantation Group. Am J Respir Crit Care Med (1994) 150(2):342–7. doi: 10.1164/ajrccm.150.2.8049813 8049813

[206] Wilkins MR Wharton J Grimminger F Ghofrani HA. Phosphodiesterase Inhibitors for the Treatment of Pulmonary Hypertension. Eur Respir J (2008) 32(1):198–209. doi: 10.1183/09031936.00124007 18591337

[207] Spratt JR Mattison LM Iaizzo PA Brown RZ Helms H Iles TL. An Experimental Study of the Recovery of Injured Porcine Lungs With Prolonged Normothermic Cellular *Ex Vivo* Lung Perfusion Following Donation After Circulatory Death. Transplant Int Off J Eur Soc Organ Transplant (2017) 30(9):932–44. doi: 10.1111/tri.12981 28493634

[208] Morrison MI Barsby JT Pither TL Griffiths CP Bates L Charlton CA. Use of Phosphodiesterase Inhibition During *Ex-Vivo* Lung Perfusion of Donor Lungs Unsuitable for Transplantation. J Heart Lung Transplant (2019) 38(4):S321. doi: 10.1016/j.healun.2019.01.1302

[209] Reffelmann T Kloner RA. Phosphodiesterase 5 Inhibitors: Are They Cardioprotective? Cardiovasc Res (2009) 83(2):204–12. doi: 10.1093/cvr/cvp170 19474180

[210] Osadchii OE Woodiwiss AJ Norton GR. Contractile Responses to Selective Phosphodiesterase Inhibitors Following Chronic Beta-Adrenoreceptor Activation. Pflugers Archiv Eur J Physiol (2006) 452(2):155–63. doi: 10.1007/s00424-005-0025-6 16369769

[211] De Silva RJ Vuylsteke A Fritchley SJ Trull AK Dunning JJ Wallwork J. APT070 Inhibits Complement Activation During *In Vitro* Cardiopulmonary Bypass. Eur J Cardio-Thorac Surg (2006) 30(1):72–6. doi: 10.1016/j.ejcts.2006.03.012 16723247

[212] Li JS Jaggers J Anderson PAW. The Use of TP10, Soluble Complement Receptor 1, in Cardiopulmonary Bypass. Expert Rev Cardiovasc Ther (2006) 4(5):649–54. doi: 10.1586/14779072.4.5.649 17081086

[213] Verrier ED Shernan SK Taylor KM Van de Werf F Newman MF Chen JC. Terminal Complement Blockade With Pexelizumab During Coronary Artery Bypass Graft Surgery Requiring Cardiopulmonary Bypass: A Randomized Trial. JAMA (2004) 291(19):2319–27. doi: 10.1001/jama.291.19.2319 15150203

[214] Kopp R Mottaghy K Kirschfink M. Mechanism of Complement Activation During Extracorporeal Blood-Biomaterial Interaction: Effects of Heparin Coated and Uncoated Surfaces. ASAIO J (American Soc Artif Internal Organs 1992) (2002) 48(6):598–605. doi: 10.1097/00002480-200211000-00005 12455769

[215] Gralinski MR Wiater BC Assenmacher AN Lucchesi BR. Selective Inhibition of the Alternative Complement Pathway by Scr1[desLHR-A] Protects the Rabbit Isolated Heart From Human Complement-Mediated Damage. Immunopharmacology (1996) 34(2-3):79–88. doi: 10.1016/0162-3109(96)00105-1 8886851

[216] Homeister JW Satoh P Lucchesi BR. Effects of Complement Activation in the Isolated Heart. Role of the Terminal Complement Components. Circ Res (1992) 71(2):303–19. 10.1161/01.res.71.2.303 1628389

[217] Hill GE Alonso A Spurzem JR Stammers AH Robbins RA. Aprotinin and Methylprednisolone Equally Blunt Cardiopulmonary Bypass-Induced Inflammation in Humans. J Thorac Cardiovasc Surg (1995) 110(6):1658–62. doi: 10.1016/S0022-5223(95)70027-7 8523876

[218] Mojcik CF Levy JH. Aprotinin and the Systemic Inflammatory Response After Cardiopulmonary Bypass. Ann Thorac Surg (2001) 71(2):745–54. doi: 10.1016/S0003-4975(00)02218-9 11235755

[219] Putnam JB Royston D Chambers AF Dunbar S Lemmer JH Norman P. Evaluating the Role of Serine Protease Inhibition in the Management of Tumor Micrometastases. Oncol (Williston Park NY) (2003) 17(10 Suppl 10):9–30; quiz 1-2. 14606367

[220] Hsia TY McQuinn TC Mukherjee R Deardorff RL Squires JE Stroud RE. Effects of Aprotinin or Tranexamic Acid on Proteolytic/Cytokine Profiles in Infants After Cardiac Surgery. Ann Thorac Surg (2010) 89(6):1843–52; discussion 52. doi: 10.1016/j.athoracsur.2010.02.069 20494037 PMC3100188

[221] Mangano DT Rieves RD Weiss KD. Judging the Safety of Aprotinin. New Engl J Med (2006) 355(21):2261–2. doi: 10.1056/NEJMc066520 17124031

[222] Lin H Chen M Tian F Tikkanen J Ding L Andrew Cheung HY. α(1)-Anti-Trypsin Improves Function of Porcine Donor Lungs During *Ex-Vivo* Lung Perfusion. J Heart Lung Transplant Off Publ Int Soc Heart Transplant (2018) 37(5):656–66. doi: 10.1016/j.healun.2017.09.019 29153638

[223] Mariscal A Nykanen A Tikkanen J Ali A Soltanieh S Duong A. Alpha 1 Antitrypsin Treatment During Human *Ex Vivo* Lung Perfusion Improves Lung Function by Protecting Lung Endothelium. J Heart Lung Transplant (2020) 39(4):S71–S2. doi: 10.1016/j.healun.2020.01.1282

[224] Prompunt E Sanit J Barrère-Lemaire S Nargeot J Noordali H Madhani M. The Cardioprotective Effects of Secretory Leukocyte Protease Inhibitor Against Myocardial Ischemia/Reperfusion Injury. Exp Ther Med (2018) 15(6):5231–42. doi: 10.3892/etm.2018.6097 PMC5996700 29904407

[225] Shibata T Yamamoto F Suehiro S Kinoshita H. Effects of Protease Inhibitors on Postischemic Recovery of the Heart. Cardiovasc Drugs Ther (1997) 11(4):547–56. doi: 10.1023/A:1007723417775 9358959

[226] Fischer UM Cox CS Jr. Allen SJ Stewart RH Mehlhorn U Laine GA. The Antioxidant N-Acetylcysteine Preserves Myocardial Function and Diminishes Oxidative Stress After Cardioplegic Arrest. J Thorac Cardiovasc Surg (2003) 126(5):1483–8. doi: 10.1016/S0022-5223(03)00792-X 14666023

[227] Xia Z Huang Z Ansley DM. Large-Dose Propofol During Cardiopulmonary Bypass Decreases Biochemical Markers of Myocardial Injury in Coronary Surgery Patients: A Comparison With Isoflurane. Anesth Analg (2006) 103(3):527–32. doi: 10.1213/01.ane.0000230612.29452.a6 16931656

[228] Eren N Cakir O Oruc A Kaya Z Erdinc L. Effects of N-Acetylcysteine on Pulmonary Function in Patients Undergoing Coronary Artery Bypass Surgery With Cardiopulmonary Bypass. Perfusion (2003) 18(6):345–50. doi: 10.1191/0267659103pf696oa 14714769

[229] Orhan G Yapici N Yuksel M Sargin M Şenay Ş Yalçin AS. Effects of N-Acetylcysteine on Myocardial Ischemia–Reperfusion Injury in Bypass Surgery. Heart Vessels (2006) 21(1):42–7. doi: 10.1007/s00380-005-0873-1 16440148

[230] Zhou H Zhou D Lu J Wu C Zhu Z. Effects of Pre–Cardiopulmonary Bypass Administration of Dexmedetomidine on Cardiac Injuries and the Inflammatory Response in Valve Replacement Surgery With a Sevoflurane Postconditioning Protocol: A Pilot Study. J Cardiovasc Pharmacol (2019) 74(2):91–7. doi: 10.1097/FJC.0000000000000698 PMC6688713 31356535

[231] de Frutos F Gea A Hernandez-Estefania R Rabago G. Prophylactic Treatment With Coenzyme Q10 in Patients Undergoing Cardiac Surgery: Could an Antioxidant Reduce Complications? A Systematic Review and Meta-Analysis. Interact Cardiovasc Thorac Surg (2015) 20(2):254–9. doi: 10.1093/icvts/ivu334 25344142

[232] Jouybar R Kabgani H Kamalipour H Shahbazi S Allahyari E Rasouli M. The Perioperative Effect of Ascorbic Acid on the Inflammatory Response in Coronary Artery Bypass Graft Surgery; A Randomized Control Trial Oronary Artery Bypass Graft Surgery. Int Cardiovasc Res J (2012) 6(1):13–7.

[233] Stanger O Aigner I Schimetta W Wonisch W. Antioxidant Supplementation Attenuates Oxidative Stress in Patients Undergoing Coronary Artery Bypass Graft Surgery. Tohoku J Exp Med (2014) 232(2):145–54. doi: 10.1620/tjem.232.145 24573122

[234] Ali- Hasan- Al- Saegh S Mirhosseini SJ Tahernejad M Mahdavi P Shahidzadeh A Karimi-Bondarabadi AA. Impact of Antioxidant Supplementations on Cardio-Renal Protection in Cardiac Surgery: An Updated and Comprehensive Meta-Analysis and Systematic Review. Cardiovasc Ther (2016) 34(5):360–70. doi: 10.1111/1755-5922.12207 27344977

[235] Hill A Clasen KC Wendt S Majoros ÁG Stoppe C Adhikari NKJ. Effects of Vitamin C on Organ Function in Cardiac Surgery Patients: A Systematic Review and Meta-Analysis. Nutrients (2019) 11(9):2103. doi: 10.3390/nu11092103 PMC6769534 31487905

[236] George TJ Arnaoutakis GJ Beaty CA Jandu SK Santhanam L Berkowitz DE. Hydrogen Sulfide Decreases Reactive Oxygen in a Model of Lung Transplantation. J Surg Res (2012) 178(1):494–501. doi: 10.1016/j.jss.2012.02.065 22464394 PMC3389187

[237] Haam S Lee S Paik HC Park MS Song JH Lim BJ. The Effects of Hydrogen Gas Inhalation During *Ex Vivo* Lung Perfusion on Donor Lungs Obtained After Cardiac Death †. Eur J Cardio-Thorac Surg (2015) 48(4):542–7. doi: 10.1093/ejcts/ezv057 25750008

[238] Wang X Wang Y Parapanov R Abdelnour E Gronchi F Perentes JY. Pharmacological Reconditioning of Marginal Donor Rat Lungs Using Inhibitors of Peroxynitrite and Poly (ADP-Ribose) Polymerase During *Ex Vivo* Lung Perfusion. Transplantation (2016) 100(7):1465–73. doi: 10.1097/TP.0000000000001183 27331361

[239] Yamada Y Iskender I Arni S Hillinger S Cosgun T Yu K. *Ex Vivo* Treatment With Inhaled N-Acetylcysteine in Porcine Lung Transplantation. J Surg Res (2017) 218:341–7. doi: 10.1016/j.jss.2017.06.061 28985871

[240] Noda K Shigemura N Tanaka Y Bhama J D’Cunha J Kobayashi H. Hydrogen Preconditioning During *Ex Vivo* Lung Perfusion Improves the Quality of Lung Grafts in Rats. Transplantation (2014) 98(5):499–506. doi: 10.1097/TP.0000000000000254 25121557

[241] Alvarez-Ayuso L Gómez-Heras SG Jorge E Guardiola JM Torralba A Granado F. Vitamin E Action on Oxidative State, Endothelial Function and Morphology in Long-Term Myocardial Preservation. Histol Histopathol (2010) 25(5):577–87. doi: 10.14670/HH-25.577 20238296

[242] Gao F Yao CL Gao E Mo QZ Yan WL McLaughlin R. Enhancement of Glutathione Cardioprotection by Ascorbic Acid in Myocardial Reperfusion Injury. J Pharmacol Exp Ther (2002) 301(2):543–50. doi: 10.1124/jpet.301.2.543 11961055

[243] Sagach VF Scrosati M Fielding J Rossoni G Galli C Visioli F. The Water-Soluble Vitamin E Analogue Trolox Protects Against Ischaemia/Reperfusion Damage *In Vitro* and *Ex Vivo*. A Comparison With Vitamin E. Pharmacol Res (2002) 45(6):435–9. doi: 10.1006/phrs.2002.0993 12162942

[244] Yu J Wang L Akinyi M Li Y Duan Z Zhu Y. Danshensu Protects Isolated Heart Against Ischemia Reperfusion Injury Through Activation of Akt/ERK1/2/Nrf2 Signaling. Int J Clin Exp Med (2015) 8(9):14793–804. PMC4658850 26628961

