# Supplementary material for: Inflammation and Oxidative Stress in the Context of Extracorporeal Cardiac and Pulmonary Support
Source: Front Immunol. 2022 Mar 4;13:831930. doi: 10.3389/fimmu.2022.831930 (PMC8931031; doi:10.3389/fimmu.2022.831930)
Supplement: Supplementary Table 1 — Highlighted studies on inflammatory and oxidative stress responses induced during extracorporeal life support, and ex-situ thoracic organ perfusion. BAL, bronchoalveolar lavage; Bcl-2, B-cell lymphoma-2; CABG, coronary artery bypass grafting; CD40, cluster of differentiation 40; Clin, clinical study; CPB, cardiopulmonary bypass; C3a, cleaved complement component C3 fragment a; C4a, cleaved complement component C4 fragment a; C5b9, cleaved complement component C5 fragment b9; ECMO, extracorporeal membrane oxygenation; Exp, experimental study; DBD, donation after brainstem death; DCD, donation after circulatory death; ESHP, ex-situ heart perfusion; ESLP, ex-situ lung perfusion; HO-1, hemoxygenase-1; IL-, interleukin; MDA, malondialdehyde; MCP-1, monocyte chemoattractant protein-1; MIF, macrophage migration inhibitory factor; MMp-2, matrix metalloproteinase-2; NADPH, Reduced nicotinamide adenine dinucleotide phosphate; PGF2, Prostaglandin F2; PMN, polymorphonuclear; SIRS, systemic inflammatory response syndrome; TNF-α, tumor necrosis factor α; TXA, tranexamic acid; VCAM-1, vascular cell adhesion molecule-1; VA-, venoarterial; VV-, venovenous [file Table_1.docx]

**Supplementary Table 1.** Highlighted studies on inflammatory and oxidative stress responses induced during extracorporeal life support, and ex-situ thoracic organ perfusion

| **Study** | **Type of ECC** | **Study design/model** | **Key findings** |
| --- | --- | --- | --- |
| Haeffner Cavaillon et al.  1989 (216) | CPB | Clin/adult patients undergone cardiac operations, n=15 | - Increased monocyte production of IL-1β 24 hours after CPB, with no significant change in IL-1β during or early after termination of CPB |
| Steinberg et al.  1991 (217) | CPB | Clin/adults undergone cardiac surgery , n=29 | - A significant elevation in the plasma IL-6, and the products of complement system activation C3a, C4a, and C5b-9 during CPB |
| Hirthler et.al.  1992 (218) | VA-ECMO | Clin/infants, n=16 | - Increased plasma IL-2, during 36 hours of ECMO in both survivors and non-survivors - Increased lipid peroxidation only briefly in survivors, but persistent in non-survivors - Only TNF-α showing a significant increase overtime during ECMO among the non-survivors |
| Ohata et al.  1995 (219) | CPB | Clin/adults undergone cardiac surgery , n=16 | - Increased plasma values of plasma IL-6, IL-8 and PMN elastase during CPB regardless of the temprature |
| Fortenberry, et al.  1996 (24) | ECMO | Clin/neonates with respiratory or cardiorespiratory failure, n=15 | - Activation of neutrophils and increased circulation IL-8 in only 15 minutes after starting of the circulation persisting for 12 hours and 24 hours respectively. - No change in TNF-α and IL-1β - Deteriorating radiographic lung injury scores during ECMO |
| McBride et al.  1996 (220) | CPB | Clin/ pediatric patients undergoing cardiac surgery, n=10 | - TNF-α and IL-1β did not change during, and after termination of CPB in either of the plasma and BAL - IL-8 and IL-10 significantly increased in plasma and BAL during and after CPB |
| Ito et al.  1997 (221) | CPB | Clin/ adults undergone elective cardiac surgery, n=26 | - An immediate increase in IL-6, IL-8 and granulocyte elastase after starting of the CPB - Higher plasma cytokines in poor oxygenation indices |
| Graulich et al.  2000 (64) | ECMO | Exp/fresh human blood, n=6 | - Increased plasma levels of neutrophil elastase, and expression of surface leukocyte adhesion molecule IL-18 without a change in the number of the leukocytes |
| Matata et al. 2000 (222) | CPB | Clin/patients +/- diabetes undergone elective coronary bypass graft surgery, n=92 | - Increasing of different markers of oxidative stress including carbonylated proteins, nitrotyrosine proteins, IL-8, and TNF-α increased in both groups during CPB - Higher marker levels in diabetic patients |
| Clermont et al.  2002 (100) | CPB | Clin/adult patients undergone elective cardiac surgery, n=23 | - Increased plasma stabilized RONS adducts after starting of CBP which remained high during the perfusion |
|  |  |  |  |
| Golej et al.  2003 (223) | VA and VVECMO | Exp/Piglet model of ARDS, n=15 | - No change in plasma cytokines during 6 hour VA-ECMO or VV-ECMO - Increased IL-1β and IL-8 in BAL in only VA group |
| Ulus et al.  2003 (224) | CPB | Clin/patients undergone cardiopulmonary bypass grafting , or mitral valve replacing surgery | - Increasing of 8-iso-PGF2 in plasma in minutes after starting of CPB - No significant increase in 15-keto-dihydro-PGF2 (inflammatory response) |
| Zimmermann et al. 2003 (225) | CPB | Clin/ adult patients undergoing elective coronary artery bypass grafting, n=5 | - Increased gene expression of IL-1β, IL-6, IL-8, and TNF-α - Return to lower than preoperative values six hours after termination of CPB |
| Chen et al.  2004 (226) | CPB | Clin RCT/ adults undergone elective cardiac operations, n=32 | - Increasing IL-8, MDA, and adhesion molecules during, and after the termination of CPB |
| Liu et al.  2005 (227) | CPB | Clin/ adult patient undergoing cardiac valve replacement surgery, n=30 | - Increased MDA, TNF-α, IL-6 and IL-10 during and after CPB |
| Christen et al.  2005 (228) | CPB | Clin/ pediatric patients undergone corrective surgery for congenital heart anomalies, n=21 | - Rapidly increasing MDA and decreasing ascorbate during CPB - Slow increasing of IL-6 and IL-8, peaking after the cessation of CPB |
| de Mendonça-Filho et al. (229) | CPB | Clin/adult patients undergoing major cardiovascular surgeries, n=93 | - Peak MCP-1 and IL-6 were observed three hours after CPB - Peak levels of MIF were reached at the end of CPB |
| Mildner et al.  2005 (230) | ECMO | Clin/infants suriviving ECMO,    n=15, and non-survivors, n=7 | - Stable molar ratio of TNF- α /solubleTNF-receptor1 in BAL in survivors and increased values in non-survivors - Undetectable IL-1β in BAL of both groups |
| Halter et al.  2005 (231) | CPB | Clin/ adult patients undergone elective cardiac surgeries, n=21 | - Increasing plasma IL-6, IL-8, and IL-10 during application of CPB - Only IL-6 levels correlated with pulmonary function |
| Amoureux et al.  2008 (232) | CPB | Clin/ adult patients undergone coronary artery re-vascularisation or valve replacement (alone or combined), n=27 | - Increased plasma IL-6, IL-8, IL10, VCAM-1, MCP-1 and , CD40 L during CPB - Increased oxidation product 1-hydroxy-3-carboxy-pyrrolidine and endogenous peroxidase activity during CPB |
| Farago et al.  2008 (233) | ESHP | Exp/mouse hearts perfused in unloaded ventricular mode, n=18 | - Increased gene expression of SOD during ESHP - Increased gene expression of NADPH oxidase, MMP-2 and MMP-9 |
| Kakishita et al. 2010 (8) | ESLP | Exp/healthy pig, n=11 | - Increasing perfusate IL-8 and TNF-α in only two hours of ESLP |
| McILwain et al.  2010 (234) | VA-ECMO | Exp/healthy Neonatal piglet, n=6 | - Increased plasma TNF-α and IL-8 in two hours after starting of CPB, before the tissue expression of these cytokines increase |
| Sadaria et al. 2011 (235) | ESLP | EXP/ initially declined donor lungs, n=7 | - Increasing expression of IL-6, IL-8, GCSF and MCP-1 in the tissue of human donor lungs during ESLP |
| Chen et al.  2014 (236) | ECMO | Exp/Healthy pigs, n=5 in ECMO and SHAM groups | - Increased plasma MDA - Decrease glutathione and SOD activity, and antioxidative capacity in only two hours, and changes reached maximum in six hours after starting of ECMO - MDA and SOD levels had returned to baseline at 24 h after starting of CPB |
| Wang et al.  2015 (237) | Neonatal ECMO | Exp/fresh human blood in roller versus diagonal pump or diagonal pump with pulsatile flow, each group n=6 | - No statistical differences for the levels of proinflammatory cytokines among the three groups |
| Passmore et al.  2016 (238) | VV-ECMO | Exp/Sheep +/- acute lung injury, smoke injury group=23, SHAM group n=20 | - Increase plasma IL-6 in injured lungs subjected to ECMO, - Increased active MMP-2 in BAL in both injured and healthy lungs subjected to ECMO, with higher values in injured lungs - Increased lung tissue macrophages in both injured control lungs, and injured lungs subjected to ECMO |
| Thangappan et al. 2016 (239) | ECMO | Clin,retrospective/adult undergone ECMO due to cardiac or respiratory failure ,n=62 | - SIRS (with infection ruled out) in 24% of the survivors |
| Ciapetti et al. 2017 (240) | ECMO | Clin retrospective/adult with ARDS as a result of H1N1 influenza, ne10 | - Decreasing plasma values of non-enzymatic antioxidants (albumin and uric acid) during the first 24 hours after the starting of ECMO |
| Masuodi et al. 2017 (241) | ESLP | Clin/DBD lungs, n=8 and DCD lungs, n=6 | - Increasing perfusate IL-6, IL-8, IL-1β and TNF-α during ESLP with higher concentrations in DCD compared to DBD |
| Andreasson et al. 2017 (73) | ESLP | Clin/initially declined donor lungs, n=16 | - The increased perfusate concentration of IL-1β and IL-8 may be used as a tool for prediction of the outcomes of ESLP |
| Vitkova et al.  2018 (67) | ECMO | Clin/newborns with severe pulmonary or cardiopulmonary compromise, n=26 | - Increased plasma IL-1β, IL-6, and IL-22, and endothelial microvesicles during application of ECMO |
| Aboelnazar et al. 2018 (27) | ESLP | Exp/human (n=5) and pig (n=32) | - Increasing perfusate IL-6, IL-8, and TNF-α during 12 hours of experimental ESLP |
| Lonati et al. 2018 (242) | ESLP | Exp/DCD rat n=15 | - Upregulation of the genes involved in inflammation, oxidative stress, and cell death including IL-6, TNF-α, HO-1, and Bcl_2_ |
| Fujii et al.  2020 (90) | ECMO | Exp, randomized/healthy rat, n=40 assigned to four groups | - Increased plasma IL-6, TNF-α, during ECMO - Higher inflammation and tissue edema in hyperoxic (PaO_2_>300 mmHg) |
| Dikme et al.  2020 (243) | CPB | Clin/ adult patients undergoing coronary bypass surgery, n=25 | - Increasing plasma oxidative stress index - Increasing mononuclear leukocytes DNA damage during CPB |
| Hatami et al. 2020 (19) | ESHP | Exp, randomized/pig hearts perfusion in loaded versus unloaded ventricle, n=14 | - Increasing perfusate pro-inflammatory cytokines including IL-6, IL-8, and TNF-α during 12 hours of ESHP - Increased tissue expression of IL-1β and TNF-α in unloaded-perfused hearts |

BAL, bronchoalveolar lavage; Bcl-2, B-cell lymphoma-2; CABG, coronary artery bypass grafting; CD40, cluster of differentiation 40; Clin, clinical study; CPB, cardiopulmonary bypass; C3a, cleaved complement component C3 fragment a; C4a, cleaved complement component C4 fragment a; C5b9, cleaved complement component C5 fragment b9; ECMO, extracorporeal membrane oxygenation; Exp, experimental study; DBD, donation after brainstem death; DCD, donation after circulatory death; ESHP, ex-situ heart perfusion; ESLP, ex-situ lung perfusion; HO-1, hemoxygenase-1; IL-, interleukin; MDA, malondialdehyde; MCP-1, monocyte chemoattractant protein-1; MIF, macrophage migration inhibitory factor ; MMp-2, matrix metalloproteinase-2; NADPH, Reduced nicotinamide adenine dinucleotide phosphate; PGF2, Prostaglandin F2; PMN, polymorphonuclear; SIRS, systemic inflammatory response syndrome; TNF-α, tumor necrosis factor α; TXA, tranexamic acid; VCAM-1, vascular cell adhesion molecule-1; VA-, venoarterial; VV-, venovenous
