# Supplementary material for: Inflammation and Oxidative Stress in the Context of Extracorporeal Cardiac and Pulmonary Support
Source: Front Immunol. 2022 Mar 4;13:831930. doi: 10.3389/fimmu.2022.831930 (PMC8931031; doi:10.3389/fimmu.2022.831930)
Supplement: Supplementary Table 2 — Pharmacologic interventions to reduce oxidative stress & inflammation in extracorporeal circulation. C3a, cleaved complement component C3 fragment a; CD11b, cluster of differentiation 11b; CK, creatine kinase; CK-MB, MB isoenzyme of creatine kinase; CPB, cardiopulmonary bypass; ECMO, extracorporeal membrane oxygenation; ESHP, ex-situ heart perfusion; ESLP, ex-situ lung perfusion; ICU, intensive care unit; IL-, interleukin;LDH, lactate dehydrogenase; MIP-1, macrophage inflammatory protein-1, MMP, matrix metalloproteinase-2; MPO, myeloperoxidase; NADPH, Reduced nicotinamide adenine dinucleotide phosphate; PAR, poly(adenosine diphosphate-ribose); PGF2, Prostaglandin F2; PMN, polymorphonuclear; POD, post-operative day; RONS, reactive oxygen and nitrogen species; SIRS, systemic inflammatory response syndrome; TNF-α, tumor necrosis factor α; VCAM-1, vascular cell adhesion molecule-1; VA-, venoarterial [file Table_2.docx]

**Supplementary Table 2.** Pharmacologic interventions to reduce oxidative stress & inflammation in extracorporeal circulation

| **Study** | **Type of ECC** | **Study Design/Model** | **Pharmacologic Intervention** | **Key Findings** |
| --- | --- | --- | --- | --- |
| **Corticosteroids** | | | | |
| Toft et al. (1997), (151) | CPB | RCT; adult patients; 8 per group | Methylprednisolone, 30mg/kg IV at induction of anesthesia | - No difference in expression of adhesion molecules or oxidative burst activity - No difference in duration of mechanical ventilation or length of ICU stay |
| Fillinger et al. (2002), (147) | CPB | RCT; adult patients; 15 per group | Methylprednisolone, 15mg/kg IV 1hr prior to surgery & 0.3mg/kg q6hr x4 | - Decreased serum IL-6 up 3 days post-op - Increased serum IL-10 60 minutes after CPB - No difference in duration of mechanical ventilation or hospital length of stay |
| Giomerelli et al. (2003), (144) | CPB | RCT; adult patients undergoing CABG; 10 per group | Methylprednisolone, 1g IV pre-op & 125mg at end of CPB | - Reduction in proinflammatory cytokines & increase in IL-10 in steroid group after CPB - Improved arterial oxygenation in steroid group - Reduced CK & CK-MB in steroid group |
| Kilger et al. (2003), (149) | CPB | RCT; adult patients; 48 control vs. 43 treated | Hydrocortisone, 100mg IV prior to induction of anesthesia, then 10mg/hr x24hr on POD1, 5mg/hr x24hr on POD2, 20mg x3 on POD3 & 10mg x3 on POD4 | - Lower post-op serum concentrations of IL-6 & lactate in steroid group - Decreased need for circulatory & ventilatory support, decrease, fewer transfusions, shorter ICU & hospital length of stay in steroid group - No difference in mortality |
| Anić et al. (2004), (142) | CPB | RCT; adult patients; 10 per group | Methylprednisolone, 30mg/kg IV prior to induction of anesthesia | - No difference in TNF-α or IL-1β between groups - Decreased post-op serum IL-6 & IL-8 concentrations in steroid group |
| Bourbon et al. (2004), (150) | CPB | RCT; adult patients; 12 per group | Methylprednisolone, 5 or 10mg/kg IV at start of CPB | - Decreased post-op serum TNF-α & IL-6 concentrations with either dose of steroid - Decreased generation of oxygen derived free radicals only with higher dose |
| Celic et al. (2004), (143) | CPB | RCT; adult patients undergoing CABG; 30 per group | Methylprednisolone, 10mg/kg IV 10min before & after CPB | - Decreased post-op serum TNF-α, IL-6 & IL-8 concentrations in steroid group - Increased post-op serum IL-10 concentration in steroid group - Increased post-op CK & CK-MB in steroid group - No difference length of hospital or ICU stay or mortality |
| Halonen et al. (2007), (148) | CPB | RCT; adult patients; 121 control vs. 120 treated | Hydrocortisone, 100mg IV given the first evening post-op, then q8hr x3 days | - Decreased incidence of atrial fibrillation post-op in the steroid group (30% vs. 48%) - No difference in rates of wound infection |
| Liakopoulos et al. (2007), (146) | CPB | RCT; adult patients undergoing CABG; 38 control vs. 42 treated | Methylprednisolone, 15mg/kg IV prior to CPB | - Decreased post-op serum TNF-α, IL-6, IL-8 & CRP concentrations in steroid group - Increased post-op serum IL-10 concentration in steroid group - Improved cardiac index & decreased troponin post-op in steroid group - No difference in clinical outcome |
| Demir et al. (2015), (145) |  |  |  |  |
| Sandha et al. (2018), (32) | ESHP (normothermic) | Non-randomized; DCD porcine model; 8 control vs. 5 treated | Methylprednisolone, 500mg added to perfusate at start of 6hr perfusion | - Decreased generation of proinflammatory cytokines TNF-α, IL-6, IL-8 & IL-1β in steroid group - Decreased myocardial edema in steroid group - No difference in perfusate troponin levels or cardiac function |
| **Antioxidants** | | | | |
| Eren et al. (2003), (199) | CPB | RCT; adult patients undergoing CABG; 10 per group | N-acetylcysteine, 100mg/kg IV 1hr prior to CPB & 40mg/kg 24hr after CPB | - Improved post-op A-a oxygen gradient & decreased malondialdehyde in treatment group - No difference in hemodynamic or pulmonary parameters - No difference in clinical outcome |
| Fischer et al. (2003), (197) | CPB | Non-randomized; canine study; 9 per group | N-acetylcysteine, 100mg/kg IV 10min prior to CPB & 20mg/kg/hr infusion until 1hr after CPB (3hr total) | - Maintenance of preload recruitable stroke work in treatment group following CPB - Decreased serum 8-isoprostane concentration in treatment group following CPB - Improved resolution of myocardial edema in treatment group following CPB |
| Orhan et al. (2006), (200) | CPB | RCT; adult patients undergoing CABG; 10 per group | N-acetylcysteine, 50mg/kg IV at induction of anesthesia | - Decreased concentration of reactive oxygen species & TNF-α in treatment group - Decreased CK-MB in treatment group at 6 & 12hr post-op - No difference in length of ICU or hospital stay or mortality |
| Yamada et al. (2017), (210) | ESLP | Non-randomized; porcine ex situ perfusion & transplantation model; 5 control vs. 6 treated | N-acetylcysteine, 50mg/kg nebulized during 2hr ESLP | - No difference in oxygenation, compliance or vascular resistance during perfusion - Decreased MPO in bronchoalveolar lavage in treatment group - Trend toward improved oxygenation in recipient |
| Gao et al. (2002), (244) | ESHP | Randomized; rat ex situ perfusion model; 10 per group | Ascorbic acid &/or glutathione monoethyl ester, 1mM added to perfusate after 30min global ischemia | - Reduction of post-ischemic injury in glutathione monoethyl ester group, enhanced with addition of ascorbic acid |
| Sagach et al. (2002), (214) | ESHP | Non-randomized; rat ex situ perfusion model; 8 control vs. 10 Trolox in perfusate vs. 7 treated with Trolox or vitamin E | Trolox, 20µmol/L added to perfusate prior to 20min global ischemia or Trolox or vitamin E, 10mg/kg PO 55min prior to induction of anesthesia | - Improved cardiac recovery in all treatment arms, less so with vitamin E - Decreased markers of oxidative stress in Trolox treatment groups |
| Alvarez-Ayuso et al. (2010), (212) | ESHP | Randomized; porcine heterotopic transplantation model; 6 control vs. 7 treated | Trolox, 0.5g/L in recirculated preservation solution & 3g given to recipient prior to implantation | - Improved systolic pressure following transplantation in treatment group - Preservation of mitochondrial & vascular morphology on histology in treatment group - Reduction in interstitial edema & inflammatory infiltrate in treatment group |
| George et al. (2012), (207) | ESLP | Non-randomized; rabbit ex situ perfusion model; 5 control vs. 7 treated | Hydrogen sulfide, 100µg/kg bolus, followed by 1mg/kg/hr infusion into perfusion circuit | - No difference in oxygenation & pulmonary artery pressures between groups - Decreased RONS after 2hr of perfusion in treatment group |
| Xia et al. (2006), (198) | CPB | RCT; adult patients; 18 per group | Propofol, 60 or 120µg/kg/min during surgery (vs. inhaled isoflurane) | - Decreased serum malondialdehyde & troponin post-op in high dose propofol group - Increased mean cardiac index 24hr post-op in high dose propofol group - Decreased ICU stay in high dose propofol group compared to isoflurane control - No difference in rates of post-op myocardial infarction |
| Jouybar et al. (2012), (203) | CPB | RCT; adult patients undergoing CABG; 20 per group | Ascorbic acid, 3g IV 12-18hr prior to surgery & again at start of CPB | - No difference in post-op serum IL-6 & IL-8 concentrations - No difference in hemodynamics or clinical outcomes |
| Stanger et al. (2014), (204) | CPB | RCT; adult patients undergoing CABG; 20 control vs. 19 vitamins vs. 19 fatty acids vs. 17 combined | n-3 polyunsaturated fatty acids, 0.5mL/kg IV 42hr & 18hr prior to surgery & 50mL 42hr post-op &/or ascorbic acid, 500mg IV, & vitamin E, 45IE IV 30min before reperfusion & 120min after | - Decreased serum peroxide concentration post-op in groups receiving vitamins - No difference in post-op atrial fibrillation |
| Yu et al. (2015), (215) | ESHP | Randomized; rat ex situ perfusion model; 8 per group | 3(3,4dihydroxyphenyl) 2 hydroxy propanoic acid (Danshensu), 1 or 10µM added to perfusate prior to 30min global ischemia | - Reduction in myocardial infarct size in treatment groups - Decreased CK & LDH concentrations in perfusate of treatment groups - Improved cardiac function after ischemia/reperfusion injury in treatment groups |
| Haam et al. (2015), (208) | ESLP | Randomized; porcine ex situ perfusion & transplantation model; 5 per group | Hydrogen, 2% during ESLP ventilation over 4hr | - Improved compliance & peak airway pressure during ESLP in treatment group - Decreased lung injury severity score & pulmonary edema in treatment group - Decreased tissue IL-6 & increased tissue IL-10 after transplantation in treatment group |
| Wang et al. (2016), (209) | ESLP | Non-randomized; rat ex situ perfusion model; 7 control vs. 6 3-AB vs. 6 MnTBAP | 3-aminobenzamide, 1mg/mL or Mn(III)-tetrakis (4-benzoic acid) porphyrin chloride, 0.3mg/mL added to perfusate | - Attenuation of increased protein carbonyls, 3-nitrotyrosine, PAR, lactate dehydrogenase & proteins in bronchoalveolar lavage in both treatment groups - Decreased weight gain & perivascular edema, & increased static compliance in both treatment groups |
| Zhou et al. (2019), (201) | CPB | RCT; adult patients undergoing valve replacement; 14 per group | Dexmedetomidine, 0.5µg/kg IV bolus, followed by 0.5µg/kg/hr during CPB | - Decreased troponin 24hr post-op in treatment group - Decreased serum TNF-α concentration, but increased serum IL-6 & IL-8 concentrations in treatment group - No difference in length of ICU stay or survival |
| **Phosphodiesterase inhibitors** | | | | |
| Yamaura et al. (2001), (173) | CPB | RCT; adult patients; 8 control vs. 6 at lower dose vs. 7 at higher dose | Olprinone, 0.1 or 0.2µg/kg/min while on CPB | - Higher dose increased serum IL-10 concentration & reduced post-op leukocytosis in treatment group - No difference in IL-6 levels - Lessened post-op surge in systemic oxygen uptake in treatment group - No difference in post-op complications |
| Hamamoto et al. (2006), (171) | CPB | Non-randomized; rat model; 5 per group | Rolipram, 40µg/kg/min for 60min while on CPB & 60min of observation | - Post-CPB increase in CD11b not seen in treatment group - Decreased in serum TNF-α & elastase concentrations in treatment group - No difference in serum IL-1β or MIP-1 |
| Osachii et al. (2006), (181) | ESHP (Langendorff) | Non-randomized; rat ex situ perfusion model; 9 per group | Isoproterenol, 50µg/kg/day IP x1mo | - Cardiac hypertrophy seen in treated group - No difference in baseline systolic function or inotropic response to calcium |
| Morrison et al. (2019), (179) | ESLP | Non-randomized; discarded human lungs; 5 lungs | Sildenafil, 10mg add to perfusate 30min after start | - Rapid development of alveolar edema in treatment group - No difference in perfusion parameters - Increased perfusate TNF-α & IL-8 concentrations in treatment group |
| Wollborn et al. (2019), (175) | VA-ECMO | Randomized; rat model; 7 per group | Rolipram, 1mg/kg/hr IV for 3hr | - No difference in serum cytokine (TNF-α, IL-1β or IL-6) concentrations - Decreased albumin extravasation in treated group - Decreased C3a levels in serum in treated group - Reduced histological injury to kidneys & lungs in treated group |
| Ustunsoy et al. (2006), (172) | CPB | Non-randomized; adult patients; 35 control vs. 40 treated | Pentoxifylline, 500mg/L added to cardioplegia solution | - Decreased serum TNF-α, IL-6 & IL-8 concentrations in treatment group - No difference in hemodynamic parameters post-op between groups |
| **Serine protease inhibitors** | | | | |
| Shibata et al. (1997), (196) | ESHP (Langendorff) | Non-randomized; rat ex situ perfusion model; 6 per group | Nafamostat mesylate, gabexate mesylate or NCO-700 added to cardioplegia or reperfusion solutions in concentrations ranging from 0.5-500µM | - Addition of any of the tested serine proteinase inhibitors improved post-ischemic cardiac recovery & reduced enzyme leakage, up to a certain dose - No beneficial effect was seen with addition to reperfusion solution & harm was even seen at some doses |
| Hsia et al. (2010), (191) | CPB | Non-randomized; pediatric patients; 22 receiving aprotinin vs. 15 receiving TXA | Aprotinin, 240mg/m^2^ BSA IV, followed by 56mg/m^2^ BSA/hr during surgery or TXA, 100mg/kg IV, followed by 10mg/kg/hr | - Decreased serum TNF-α, MMP-8 & MMP-9 concentrations post-op in aprotinin group - Increased serum IL-10 concentration post-op in TXA group |
| Lin et al. (2018), (193) | ESLP (normothermic) | Randomized; porcine ex situ perfusion model; 6 per group | α_1_-anti-trypsin, 3mg/mL added to perfusate | - Decreased pulmonary arterial pressure, pulmonary vascular resistance, airway pressure & pulmonary edema in treatment group - Decreased perfusate concentrations of IL-1α & IL-8 in the treatment group |
| Prompunt et al. (2018), (195) | ESHP (Langendorff) | Non-randomized; mouse ex situ perfusion model; 6 per group | Recombinant human secretory leukocyte protease inhibitor, 400 or 1000ng/mL added to perfusion solution | - Decreased infarct size following myocardial ischemia reperfusion at both doses |
| Mariscal et al. (2020), (194) | ESLP | Randomized; discarded human lungs; 8 pairs divided into control & treatment | α_1_-anti-trypsin, dose not reported | - Improved oxygenation & compliance & lower pulmonary arterial pressure & pulmonary edema in treatment group |
| Hill et al. (1995), (188) | CPB | RCT; adult patients; 8 per group | Methylprednisolone, 1g IV 5min prior to CPB or aprotinin, 140mg IV loading dose, followed by 35mg/hr infusion while on CPB | - Decreased serum TNF-α concentration 30min after CPB in both treatment groups - Decreased serum CD11b concentration during & after CPB in both treatment groups - No difference in inflammatory markers between treatment groups |
| **Statins** | | | | |
| Chee et al. (2017), (165) | CPB | RCT; adult patients; 15 per group | Atorvastatin, 80mg PO daily x2wks prior to surgery | - Decreased post-op troponin & urine neutrophil gelatinase-associated lipocalin in treated group - Increased serum IL-8 concentration at baseline & immediately post-op - No difference in clinical outcome |
| Liu et al. (2017), (170) | ESHP (Langendorff) | Randomized; rat ex situ perfusion model; 8 per group (except 5nmol/L group where n=16) | Rosuvastatin, 1, 5, 10, 25 or 50nmol/L in perfusion solution after period of coronary artery occlusion | - Reduction of infarct size & improved cardiac function with doses of 5 or 10nmol/L - Loss of effect & harm with doses >10nmol/L |
| Vélez et al. (2020), (169) | ESHP (Langendorff) | Randomized; rat ex situ perfusion model; 6 per group | Rosuvastatin, 3µM added to perfusion solution prior to 25min of global ischemia | - Reduction of myocardial infarct size & improved cardiac function in treatment group - Improved mitochondrial function in treatment group with decreased lactic acid build up & increased glycogen consumption |
| **Complement inhibitors** | | | | |
| Gralinski et al. (1996), (186) | ESHP | Non-random; rabbit ex situ perfusion model; 13 control vs. 10 per treatment group | Soluble complement receptor type 1 or truncated version, 20µM added to perfusate | - Improved cardiac parameters (coronary perfusion pressure, left ventricular end diastolic pressure) in both treatment groups - Prevention of classical & alternative pathway mediated hemolysis by full version |
| Verrier et al. (2004), (184) | CPB | RCT; adult patients undergoing CABG; 1546 control vs. 1553 treated | Pexelizumab, 2mg/kg IV, followed by 0.05mg/kg/hr x24h post-op | - Decreased likelihood of patients dying or experience myocardial infarction in treatment group - No difference in risk of mortality alone |
| De Silva et al. (2006), (182) | CPB | Non-randomized; in vitro study of human blood in CPB circuit; 4 control vs. 5 treated | APT070 (micrococept), 50µg/mL concentration in circulated blood | - Decreased C3a & sC5b-9 concentration (complement activation) in treatment group - Decreased CD11b concentration (neutrophil stimulation in treatment group |

C3a, cleaved complement component C3 fragment a; CD11b, cluster of differentiation 11b; CK, creatine kinase; CK-MB, MB isoenzyme of creatine kinase; CPB, cardiopulmonary bypass; ECMO, extracorporeal membrane oxygenation; ESHP, ex-situ heart perfusion; ESLP, ex-situ lung perfusion; ICU, intensive care unit; IL-, interleukin;LDH, lactate dehydrogenase; MIP-1, macrophage inflammatory protein-1, MMP, matrix metalloproteinase-2; MPO, myeloperoxidase; NADPH, Reduced nicotinamide adenine dinucleotide phosphate; PAR, poly(adenosine diphosphate-ribose); PGF2, Prostaglandin F2; PMN, polymorphonuclear; POD, post-operative day; RONS, reactive oxygen and nitrogen species; SIRS, systemic inflammatory response syndrome; TNF-α, tumor necrosis factor α; VCAM-1, vascular cell adhesion molecule-1; VA-, venoarterial
